# Notch Signaling Maintains Neural Rosette Polarity

**DOI:** 10.1371/journal.pone.0062959

**Published:** 2013-05-10

**Authors:** Heather Main, Jelena Radenkovic, Shao-bo Jin, Urban Lendahl, Emma R. Andersson

**Affiliations:** Department of Cell and Molecular Biology, Karolinska Institutet, Stockholm, Sweden; Instituto de Medicina Molecular, Portugal

## Abstract

Formation of the metazoan body plan requires a complex interplay of morphological changes and patterning, and central to these processes is the establishment of apical/basal cell polarity. In the developing nervous system, apical/basal cell polarity is essential for neural tube closure and maintenance of the neural stem cell population. In this report we explore how a signaling pathway important for nervous system development, Notch signaling, impacts on apical/basal cell polarity in neural differentiation. *CSL^−/−^* mouse embryos, which are devoid of canonical Notch signaling, demonstrated a neural tube phenotype consistent with cell polarity and convergent extension defects, including deficiencies in the restricted expression of apical polarity markers in the neuroepithelium. *CSL^−/−^* mouse embryonic stem (ES) cells, cultured at low density, behaved as wild-type in the establishment of neural progenitors and apical specification, though progression through rosette formation, an *in vitro* correlate of neurulation, required *CSL* for correct maintenance of rosette structure and regulation of neuronal differentiation. Similarly, acute pharmacological inhibition of Notch signaling led to the breakdown of neural rosettes and accelerated neuronal differentiation. In addition to functional Notch signaling, rosette integrity was found to require actin polymerization and Rho kinase (ROCK) activity. Disruption of rosettes through inhibition of actin polymerization or ROCK activity, however, had no effect on neuronal differentiation, indicating that rosette maintenance is not a prerequisite for normal neuronal differentiation. In conclusion, our data indicate that Notch signaling plays a role not only in differentiation, but also in organization and maintenance of polarity during development of the early nervous system.

## Introduction

Development of the central nervous system (CNS) relies on intricate developmental programs to control the proliferation and differentiation of multiple cell types from neuroepithelial progenitors. Differentiation processes for establishing cellular diversity are coupled to complex morphological transitions. The CNS forms from the neural plate, a thickened pseudostratified epithelium, which, in mammals, bends and fuses to form the anterior neural tube in a process known as primary neurulation [1]. Disturbances in neurulation lead to grave developmental defects including anencephaly and myelomeningocele (spina bifida), while disturbances in proliferation, differentiation or synaptogenesis are associated with any number of disorders including hyperactivity, learning disabilities, autism, schizophrenia, depression and cancer [2].

Central to formation of the neural tube and nervous system patterning is the establishment of apical/basal and planar cell polarity. In apical/basal polarity, individual cells asymmetrically partition cellular components to collectively identify the apical and basal side of the cell, whereas in the case of planar cell polarity, lateral faces of the epithelial sheet of cells are defined. Neural plate bending and tube closure requires planar cell polarity/convergence extension processes [3], which act in parallel with regulation of the actin cytoskeleton by Rho kinase (ROCK) and its activator RhoA for apical constriction and hinge point formation [4]–[10]. How polarity links to intracellular cell fate signaling mechanisms is particularly interesting in neuronal differentiation where the apical/basal location of cells within the multilayered neuroepithelium correlates with their differentiation status [11].

The developmental program linking neural differentiation with morphological transitions can be studied in vitro using Embryonic Stem (ES) cell neural rosette formation, considered an vitro correlate of neural tube formation [12], [13]. Neural rosettes form autonomously during both embryoid body and monolayer ES cell neural differentiation in the absence of non-neural cell types [14], [15]. Neural rosettes and the early neural tube both display an inner apical, undifferentiated identity and an outer basal, more differentiated identity [12], [13] as well as the expression and apical/basal organization of several markers, including Sox1, CD133/prominin, Pax6, Brain lipid binding protein (BLBP), Nestin, aPKCζ and TuJ1 [12], [13], [16]. Overexpression of the ‘stemness’ gene USP9X or the polarity gene Crumbs2 in ES cells has been shown to enhance neural progenitor proliferation and the number of rosette structures [17], [18], suggesting a link between polarity, proliferation and rosette morphology.

The idea that cell polarity works in conjunction with major cell signaling pathways to regulate differentiation is an emerging concept. The Notch signaling pathway is an attractive candidate for exploration of this crosstalk, as *CSL^−/−^* embryos, devoid of canonical Notch signaling, fail to complete neurulation [19], a sign of defective polarity. Other mice lacking Notch components have also been reported to manifest a wavy or kinked neural tube, neural tube closure defects and/or anterior-posterior body axis shortening [20]–[28], phenotypes generally associated with cellular polarity mutants such as *Shroom* [*29*], *Vangl* [*30*], *Dvl* [31] or *Wnt5a* [*32*] knockouts. Notch has been shown to play a key role in maintaining neural progenitors by regulating asymmetric cell divisions that depend on the partitioning of components in the apical/basal axis [33].

Notch signaling proceeds via interaction between membrane-tethered Notch ligands and receptors on contacting cells. Following ligand binding, the Notch receptor undergoes successive proteolytic cleavages to release the Notch intracellular domain (Notch ICD). Notch ICD then translocates to the nucleus, where it binds to the DNA-binding protein CBF-1/Suppressor of hairless/Lag-1 (CSL, a.k.a. RBPJ-κ), converting it from a transcriptional repressor to an activator. While Notch signaling is well defined as a mediator of cell fate decision processes [34], it has more recently been linked to cell polarity control. In zebrafish, an apical/basal Notch gradient is required for retinal cell type specification [35] and in zebrafish neural development, non-canonical Notch maintains neuroepithelial polarity downstream of the crumbs inhibitor Moe [36]. In mammalian neural development, Notch is positively regulated by the PAR complex proteins Pard3 and aPKC, promoting apical neuroepithelial identity [37], [38], and is negatively regulated by the basolateral promoting protein Pard1, inducing neuronal differentiation [39]. Further, Hes genes, which are immediate Notch downstream target genes, are required for neuroepithelial integrity and radial glial cell formation [40]. Treatment of ES cell-derived neural rosettes with γ-secretase inhibitors, which block proteolytic cleavage of the Notch receptor [41]–[43], has been noted to affect rosette maintenance [12], [16], [36]. Collectively, these data suggest Notch signaling is linked to apical/basal polarity in maintenance of neural tube and ES cell-derived neural rosette morphology.

In this report, we address the relationship between neuronal differentiation, cell polarity and Notch signaling. We show that *CSL^−/−^* mice, lacking canonical Notch signaling, exhibit varied degrees of neurulation defects concurrent with altered apical/basal polarity in the developing neural tube. In ES-cell derived neural rosettes, we show that Notch signaling is absolutely required for maintenance of neural rosettes, though it is not required for induction of apically defined neural progenitors. Loss or inhibition of Notch signaling drives break-down of apical polarity and accelerates neuronal differentiation, while inhibition of actin polymerization or ROCK activity eroded rosettes but did not seem to affect differentiation, suggesting that polarity and regulation of neuronal differentiation are independent processes. These data shed new light on the roles of Notch in regulating not only differentiation, but also the formation and maintenance of polarized structures in the developing nervous system.

## Materials and Methods

### Animal Maintenance and Embryo Collection

*RBPJκ^loxP/loxP^* mice [44] were bred with CMV-cre mice to obtain *RBP^+/−^* mice (herein referred to as *CSL^+/−^*). *CSL^+/−^* mice were maintained on a C57bl6 background and were mated overnight to obtain *CSL^−/−^*, *CSL^+/−^* and *CSL ^+/+^* embryos. Mice were housed and bred in accordance with the approval of the local ethics committee (Stockholm’s Norra Djurförsöksetiska nämnd). All animal experiments were approved by Stockholms Norra djurförsöksetiska nämnd. Noon of day of plug was taken as embryonic day (E) 0.5. Embryos were dissected out in PBS, fixed in 4% paraformaldehyde 4 hours to overnight at 4°C, and then washed several times in phosphate buffered saline (PBS). Embryos were transferred to 30% sucrose in PBS, rocked overnight, embedded in OCT (TissueTek), and frozen on dry ice for cryostat sectioning and immunohistochemistry. Serial transverse 14-µm sections were collected on SuperFrost glass slides on a cryostat.

### Genotyping

The *CSL* mutant allele has been described previously [44]. DNA was extracted from ear or embryonic tissues by first boiling at 95°C for 20–40 minutes in 50–200 µl of 25 mM NaOH/0,2 mM EDTA and then an equal volume of 40 mM Tris HCl pH 5 was added to neutralize the sodium hydroxide. 3 μl of this solution was used for PCR. The forward primer CSL-F 5′-ACC AGA ATC TGT TTG TTA TTT GCA TTA CTG-3′ and two reverse primers CSL-R1 5′-TAA TGC ACA CAA GCA TTG TCT GAG TTC-3′ and CSL-R2 5′-ATG TAC ATT TTG TAC TCA CAG AGA TGG ATG-3′ were used to detect the wild-type and mutant alleles.

### Cell Culture

ES cell lines used were the *CSL^+/−^* and *CSL^−/−^* ES cells (kind gifts from Timm Schroeder and Tasuku Honjo [19]) and *CSL^−/−^* rescued with pCAG, pCAG-CSL and NERT^ΔOP^ (kind gift from Timm Schroeder and Ursula Just [45]) and wild-type Sox1-GFP 46C and Tau-GFP ESC (kind gift from Austin Smith [14]). ES cells were maintained on gelatin (Sigma) coated dishes (Corning) in knockout DMEM medium (Gibco) supplemented with 5% ESC tested FCS (Sigma), 5% KSR (Gibco), glutamine (Gibco), non-essential amino acids (Gibco), beta-mercaptoethanol and LIF (Millipore) at 5% CO_2_ at 37°C. Cells were passaged the day prior to initiation of differentiation. The following day cells were plated in N2B27 media on gelatin at 1–2×10^5^ cells per well in 6-well plates or 0.25–0.5×10^5^ cells per well in 24-well plates (Corning). N2B27 medium consists of a 1∶1 ratio of DMEM/F12 (Gibco) and neurobasal media (Gibco) supplemented with 0.5% N2 (Gibco) 0.5% B27 (Gibco), 0.1 mM beta-mercaptoethanol and 1× glutamine (Gibco). Medium was changed every day.

### Neurosphere Derivation

*CSL^+/−^* or *CSL^−/−^* cells were grown, as described above, as monolayers for 7 days. At day 7, cells were collected and resuspended in growth medium as described previously [46]. Neurospheres were counted after 5 days of growth, using trypan blue to exclude clusters of dead cells. For assessing the effect of inhibitors on neural stem cell potential, *CSL^+/−^* cells were grown until day 7 in monolayers, and then treated overnight with DAPT, Cytochalasin D, or Y27632 at the concentrations detailed below. After overnight treatment, cells were collected as above for neurosphere growth and also quantified after 5 days.

### Inhibitor Treatments

Concentrations of inhibitors used are shown in Table 1. Inhibitor treatments were performed overnight unless otherwise indicated.

**Table 1:** Inhibitors.

Inhibitor	Company	Catalogue #	Final concentration
DAPT	Calbiochem	565784	2.5 ng/mL
Cytochalasin D	Sigma Aldrich	C8273	50 nM
Y27632	Calbiochem	688001	200 uM

### Q-PCR Analysis

RNA was isolated using QIAGEN RNeasy mini RNA extraction kit and cDNA was produced using Invitrogen reverse transcriptase, according to the manufacturer’s protocol. Primer sequences used were from published papers or designed with Primer express, for sequences, please see Table 2.

**Table 2:** qPCR Primer Sequences.

Gene	Forward -5′–3′	Reverse-5′–3′
CD133	TTAAACCAGGAGCTGCCCAA	CAGCAAGCCCAGGAAAAAGA
Pax6	TGGCAAACAACCTGCCTATG	TGCACGAGTATGAGGAGGTCT
BLBP[50]	GGGTAAGACCCGAGTTCCTC	ATCACCACTTTGCCACCTTC
Pard3	GTCACATTTTCGTGCATGCC	GCACTTTAGCAACCCAGCCTT
Shroom3	GTGACCTCGACGATCCAAAAG	CGGATCAAATGGCTGCACT
PKCz	CGGGACCTAAAACTGGACAA	GATTTCGGGGGCGATATAGT
Sox1	AAAACCCCAAGATGCACAACTC	TCTTGAGCAGCGTCTTGGTCT
UBC2[78]	AGGAGGCTGATGAAGGAGCTTGA	TGGTTTGAATGGATACTCTGCTGGA

All Q-PCR experiments were performed using SYBR green mastermix (Applied Biosystems) and 5 ng/μl of forward and reverse primers and analyzed in real time using 7500 system SDS Q-PCR software (Applied Biosystems). All statistics are t-tests comparing *CSL^−/−^* and *CSL^+/−^* samples from the same day of differentiation.

### Immunohistochemistry on Neural Tube and Assessment of Apical Markers

Sections on slides were outlined with a PAP-pen and then rehydrated in PBS for 5 min. Slides were then blocked with 5% donkey serum (DS) in phosphate-buffered saline (PBS) with 0.1% Triton-X (PBST) for 1 hr. Primary antibodies were diluted in 5% DS/PBST overnight at 4°C. Slides were then washed three times in PBS, fifteen minutes for each wash, and secondary antibodies, diluted in 5% DS/PBST, were applied for 1 hr at room temperature. The slides were then washed again, three times for fifteen minutes, and counterstained with DAPI and/or phalloidin (Invitrogen, 5 μL in 200 μL, 10 min) and washed twice more. Phalloidin was reconstituted according to the manufacturer’s instructions. For antibody information please see Table 3.

**Table 3:** Antibody information.

Antibody	Company	Catalogue number	Dilution
BLBP	Abcam	Ab32423	1∶500
CD133	eBioscience	14-1331-80	1∶50 (1∶500 sections)
N-cadherin	Santa Cruz	SC7939	1∶200
Nestin 130	[79]		1∶1000
Notch1-C20	Santa Cruz	Sc-6014	1∶500
Pard3	Millipore	07–330	1∶500
Pax6	Millipore	Ab2237	1∶500
PKCz	Abcam	Ab59412	1∶200
Secondary antibodies	Molecular Probes		1∶200
Sox2	Santa Cruz	SC1730X	1∶200
Sox2	Millipore	Ab5603	1∶500
Sox3	Kind gift from Prof Jonas Muhr		1∶1000
TuJ1	Promega	G712A	1∶1000
ZO1	Invitrogen	402200	1∶500 (1∶50 sections)

Images were acquired on a Zeiss LSM 510 confocal microscope, using the same settings for all embryos.

### Immunohistochemistry on ES Cells

Adherent cells were fixed for 10 min at room temperature with 4% PFA, washed twice with PBS and left in PBS overnight at 4°C. Cells were blocked with 5%DS/PBST for 1 hour and stained overnight with primary antibodies diluted in 5%DS/PBST. Cells were then washed three times for 5 min in PBS and secondary antibodies, diluted in 5%DS/PBST were applied for 1 hr at RT. For further antibody information, please see Table 3. Cells were washed three times for 15 min in PBS and counterstained with 1∶100 phalloidin (Invitrogen) and/or 1∶1000 DAPI, for 30 minutes at room temperature. Samples were washed three times for 5 min with PBS and all images were acquired with a 10× objective on a Zeiss Axiovert 200M or Zeiss Observer Z1 inverted microscope using Openlab 3.1.7 software. Differentiation of ES cells on glass for confocal analysis was performed using poly-ornithine/laminin coating. Culture slides (BD Falcon) were coated for 2 hours with 0.01% polyornithine (SIGMA), washed twice for 5 min with PBS and then coated overnight with 4 ug/mL laminin (Invitrogen). Slides were mounted in glycerol/PBS 9∶1 and all confocal images were acquired on a Zeiss LSM 510 (Zeiss, Germany), with Zeiss software.

### Assessment of Rosette Subtype

ES cell differentiations were stained with Phalloidin and DAPI. Images were acquired on a Zeiss Axiovert 200 M at 10× or 20×. Lumen size, colony size and DAPI intensity were measured using Adobe Photoshop (Adobe Systems Inc.). Rosette subtype was assessed using the following formula:
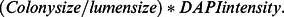



Using these calculations, rosettes fell neatly into two categories, with lower values reflecting small lumens and low cell density (abbreviated to “small-lumen rosettes”), and higher values reflecting large lumens and high cell density (abbreviated to “large-lumen rosettes”).

### Live Imaging

Live imaging was performed in 24 well tissue culture plates (Corning) overnight at 37°C. Images were acquired with a 10× objective every 20 min using the Image Xpress Micro (Molecular Devices) and data was collected and analyzed using Meta Express software.

### Flow Cytometry

24 well samples were trypsinised for 2 minutes and resuspended in 1 mL 10% fetal calf serum/PBS. Samples were analysed with a FACSCalibur and CellQuest Pro software.

### Statistics

Student t-tests were performed for comparing two groups, and ANOVA with repeated measures and Dunnett’s correction for multiple comparisons were used for comparing multiple groups. 2-way ANOVA, with Sidak’s multiple comparison test, were used to compare two groups over several days (eg development of rosettes day 1–8). Analyses were performed using Excel (Microsoft Offic) or Graph Pad Prism 6 (GraphPad Software). Significance is indicated with *for p<0.05, **p<0.01, ***p<0.001 and ****<0.0001. Error bars represent the mean ± SD of three independent experiments unless otherwise indicated. triplicate, error bars indicate standard deviation: ***significant difference at p<0.001; **significant difference at p<0.01; *significant difference at p<0.05.

## Results

### *CSL^−/−^* Embryos Display Neural Tube Defects Consistent with Planar Cell Polarity and Apical/Basal Defects

**Figure 1:**
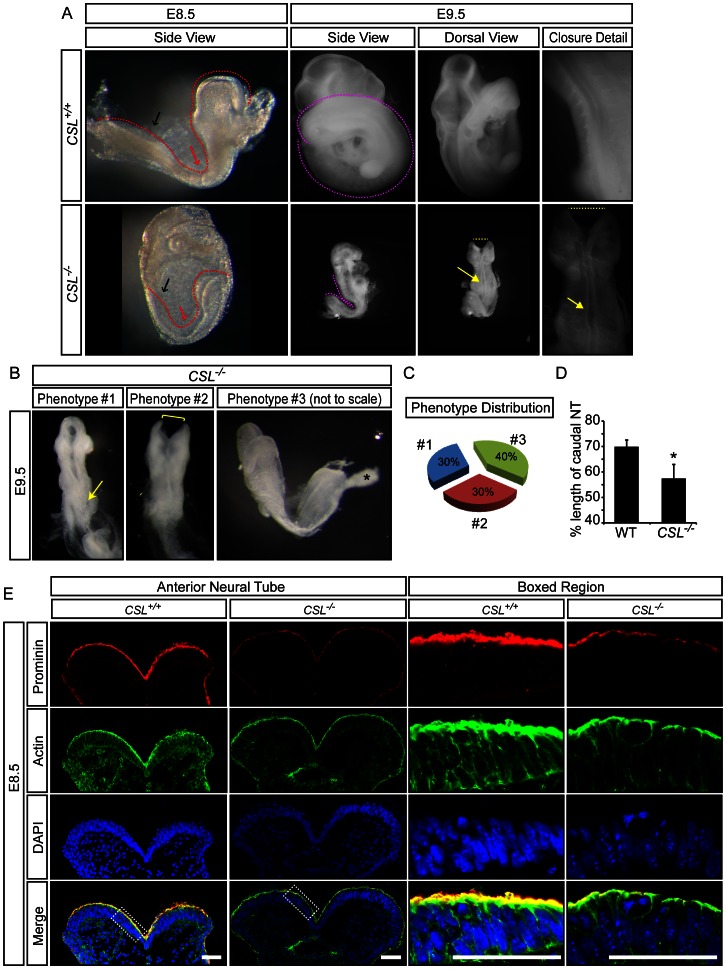
Notch signalling is required for appropriate neurulation. (A) At E8.5, *CSL^−/−^* embryos are somewhat developmentally delayed and display a shorter anterior-posterior axis (red dotted line) than WT littermates, which is particularly evident in the caudal region (black arrow). At E9.5, *CSL^−/−^* embryos are still smaller than wild-type littermates, and have usually failed to turn. *CSL^−/−^* embryos display neural tube defects such as an open anterior neural tube (yellow dotted lines), a kinked neural tube closure line in the thoracic levels (yellow arrow) and convergent extension defects with a shortened caudal anterior-posterior axis (pink dotted lines). (B, C) At E9.5, *CSL^−/−^* embryos are obtained at approximately Mendelian ratios (27.78%, 10 out of 36) and these manifest three different levels of penetrance of the phenotype, present in approximately equal frequencies (#1 = 30%, #2 = 30%, #3 = 40%). The mildest *CSL^−/−^* phenotype (#1) manifests as a slight developmental delay, disrupted caudal development and an undulating neural tube closure line (yellow arrow). Phenotype #2 includes an equivalent developmental delay as seen in Phenotype #1, but these embryos have an open neural tube in both the anterior (yellow bracket) and posterior regions. Phenotype #3 is grossly developmentally delayed, resembling approximately E8.5, and has not yet undergone neurulation. The images for wild-type, Phenotype #1 and #2 were taken at the same magnification, while Phenotype #3 was taken at a larger magnification as it was much smaller. (D) Convergent extension was quantified in E9 embryos by dividing the length of the caudal neural tube (from the caudal hindbrain to the tip of the tail), in which convergent extension is the most pronounced, by the length of the entire neural tube (from anterior forebrain to the tip of the tail). (WT N = 4, *CSL^−/−^* N = 3, p = 0.047). (E) Coronal sections of E8.5 anterior neural tube show decreased apical actin and CD133 staining in *CSL^−/−^* embryos. Scale bar is 50 μm. Please see Fig. S1 for additional markers at other stages.

To explore if Notch signaling contributes to neural tube morphogenesis *in vivo*, we analyzed mouse embryos genetically deficient for *CSL*. At embryonic day 8.5 (E8.5), *CSL^−/−^* embryos begin to show phenotypic abnormalities [19]. At E8.5 and E9.5 CSL−/− embryos display a shortened anterior-posterior axis (Fig. 1A), but are obtained at approximately the expected Mendelian proportion (28% actual versus 25% expected, N = 36). However, within this population, there was a variation in the severity of the phenotype, with three main classes of phenotype at E9.5 (Fig. 1B, C). The least severely affected embryos (Phenotype #1) were slightly developmentally delayed (Theiler stage, TS, 14), compared to wild-type embryos which were TS15 (Fig. 1C). Their caudal development was impaired, but the neural tube was appropriately closed anteriorly. *CSL^−/−^* embryos displaying an intermediate phenotype (Phenotype #2) were TS13/TS14 with disrupted caudal development and an open neural tube anteriorly. The most severely affected *CSL^−/−^* embryos (Phenotype #3) were TS11-12 (equivalent to ca E8.5), and had not undergone neurulation. *CSL^−/−^* embryos of phenotype #1 or #2 displayed an undulating neural tube closure line in the thoracic region, typical of planar cell polarity/convergence extension-related neural tube defects (yellow arrows in Fig. 1C and magnified in Fig. 1D). In sum, all *CSL^−/−^* embryos displayed neural tube defects of varying severity, and 70% had failed to undergo complete neurulation by E9.5.

In light of the morphological findings, and in particular the undulating neural tube phenotype, we next examined apical polarization in the neural tube at E8.5–E8.75, a time at which neurulation has been completed in the thoracic, but not anterior, region of wild-type mice. The apical domain is characterized by accumulation of Prominin1 (CD133), the polymerized form of globular-actin, filamentous-actin (F-actin), and ZO-1 [47], [48]. As both planar cell polarity and convergence-extension require correct apical/basal polarity, we analyzed the apical expression of Prominin1 (CD133), ZO-1, and F-actin in sections of the *CSL^−/−^* and wild-type neural tube in the anterior and thoracic region. Of four embryos analyzed, apical Prominin and actin were reduced in the anterior neural tube (Fig. 1E) of two stage-matched *CSL^−/−^* embryos, when compare to their littermate wild-types, while the other two *CSL^−/−^* embryos were significantly developmentally delayed and could not be compared (apical markers decrease with development stage [49]). Two of three *CSL^−/−^* embryos analyzed at E8.75 showed a decrease in Prominin, ZO-1 and F-actin in the thoracic region (Fig. S1), while the third was developmentally delayed and did not show a decrease in these markers. Collectively, these data indicate that *CSL*-deficient mouse embryos fail to undergo normal neurulation and are defective in their ability to properly initiate and/or maintain apical neuroepithelial polarity.

### *CSL*-deficient ES Cells Undergoing Neural Differentiation Display Polarity Defects

**Figure 2:**
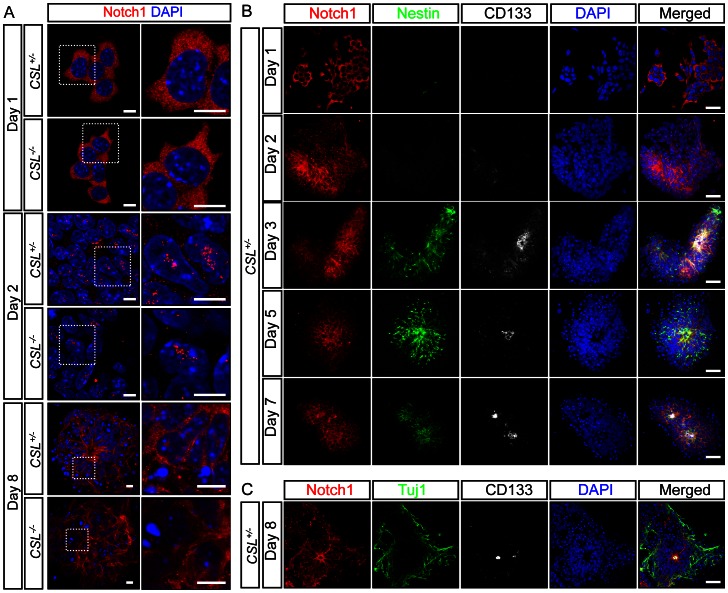
Notch1 is expressed in ES cells undergoing neural differentiation. (A) Notch1 is detected homogenously throughout the cytoplasm of ES cells undergoing neural differentiation at Day 1, using an antibody specific for the C-terminus of Notch1 (detecting cleaved and full-length Notch1). Notch1 translocates to the nucleus on Day 2 or 3 of neural differentiation, and can be found in two or three specific nuclear loci. Notch1 is detectable until Day 8. (B) During differentiation Notch1 expression is enriched in Nestin+ neural progenitor cells from Day 3, and is maintained until Day 8. (C) Notch1 expression does not coincide with Tuj+ neurons, shown here at Day 8. In (A) scale bar is 10 μm, in (B) and (C) scale bar is 40 μm. For separate channels please see Fig. S3.

**Figure 3:**
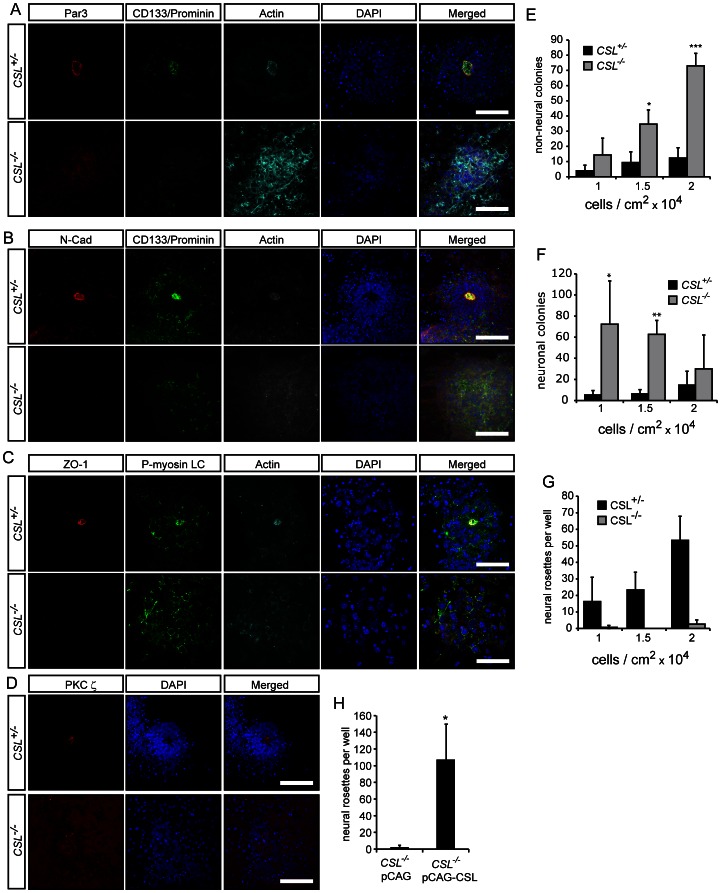
Notch signalling is required for the presence of neural rosettes at Day 8 of differentiation. (A) *CSL^+/−^* and *CSL^−/−^* ES cells were differentiated for 8 days under neural differentiation conditions. *CSL^−/−^* differentiations do not contain rosettes, as assessed by staining for rosette lumen-specific markers (A) Par3 (Pard3)/CD133 (Prominin)/Actin, (B) Zo-1/phosphorylated myosin light chain (P-MLC)/Actin, (C) N-Cadherin/CD133Actin, and (D) PKCξ stainings. (E–F) Seeding density (1, 1.5 and 2×10^4^ cells/cm^2^) contributes to the numbers of non-neural (E) and neuronal (F), colonies, per well, obtained from *CSL^−/−^* ES cells but no seeding density rescued rosette formation (G) after 8 days of neural differentiation. (H) The lack of rosettes in *CSL^−/−^* differentiations is *CSL*-specific, and not cell-line specific, as the rosette defect seen in *CSL^−/−^* ES cells can be rescued by stable re-introduction of *CSL*. Scale bars in all panels are 50 μm. For separate channels please see Fig. S4.

In order to investigate the requirement for *CSL* in neural induction and polarity maintenance we utilized the cell autonomous nature of ES cell-derived neural rosette formation, which allows analysis and manipulation of neural tube-like formation processes in the absence of secondary embryonic effects. Briefly, ES cells were plated on gelatin in N2B27 medium for 8 days [14]. These conditions stimulate neural induction within 3 days, peaking around day 6, and neural rosette structures appear within 4 to 6 days [14]. Neural rosettes and the neural tube share several characteristic traits including proliferative neural stem cells surrounding an apical lumen, delineated by specific markers, and more differentiated cells at the basal periphery (Fig. S2). Notch1 was expressed by the cells throughout this differentiation protocol, and was found primarily in the cytoplasm at day 1, but rapidly trans-located to the nucleus at day 2, in both *CSL^+/−^* and *CSL^−/−^* cells (Fig. 2A and Fig. S3). Through day 3–8 Notch1 expression became restricted to specific cells, and was found at the center of neural rosettes (Fig. 2A, B), in neural stem cells visualized with Nestin staining (Fig. 2B), but not in neurons visualized with Tuj1 staining (Fig. 2C). Neural rosettes, identified by luminal accumulation of Par3/CD133/Actin/N-Cadherin/ZO-1 or phosphorylated myosin light chain (P-MLC), were frequently observed in control (*CSL^+/−^*) differentiations at 8 days of neural differentiation (Fig. 3A–D and Fig. S4). However, in *CSL^−/−^* ES cell differentiations, neural rosettes were not seen, and instead clusters of cells lacking apically accumulated markers or lumens were present. Further, the appearance of control and *CSL^−/−^* cultures was quite distinct. The culture surface of *CSL^−/−^* differentiations was covered with large, flat cells indicative of non-neural differentiation [50] and extensive neurite networks stretched between non-polarized, rounded *CSL^−/−^* neural colonies (Fig. S5).

Cell plating density is known to affect neural differentiation in vitro, with high-density overgrown cultures undergoing BMP-induced non-neural differentiation at the expense of neural cells [51]. We therefore addressed whether lower density cultures would rescue the loss of rosettes in *CSL^−/−^* neural differentiations. Cells were plated at 1/1.5/2×10^4^ cells per cm^2^ and compared at day 8. All densities support efficient *CSL^+/−^* neural differentiation. High density culture of *CSL^−/−^* cells favored non-neural colonies, spreading as monolayers of large flat cells (Fig. 3E), whereas lower seeding densities favored neuronal differentiation (colonies of cells with neurites) (Fig. 3F). Rosette formation was severely reduced at all seeding densities of *CSL^−/−^* differentiation (Fig. 3G). The seeding density did not have a pronounced effect on neuronal or non-neural differentiation in *CSL^+/−^* wild-type cultures, although the number of neural rosettes increased at higher cell densities (Fig. 3G). The results extend previous reports showing that Notch promotes neural differentiation while suppressing mesoderm differentiation from pluripotent cells [50], [52], [53]. Our experiments were therefore performed at low density, at which the lack of Notch signaling has very little effect on non-neural differentiation. To further verify the role of Notch in rosette development, we confirmed that reintroduction of *CSL* to *CSL^−/−^* ES cells [54], rescued rosette numbers (Fig. 3H), confirming that loss of *CSL*-mediated Notch signaling is responsible for the observed reduction in rosettes.

### Notch Receptor Activity is Required for Rosette Integrity and Modulates Rosette Number

**Figure 4:**
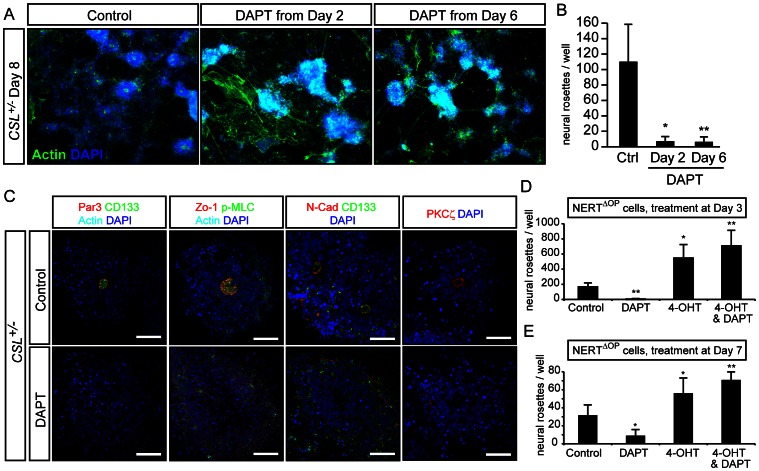
Notch is required for rosette maintenance. (A, B) Day8 *CSL^+/−^* ES cell neural differentiations, treated with the γ-secretase inhibitor DAPT from Day 2 or Day 6, display a drastic reduction in rosette number, visualized with staining for actin and DAPI (A), quantified in (B). (C) Acute treatment of *CSL^+/−^* cells with DAPT for 16 hours between Day 7 and Day 8 leads to a break-down of existing rosettes, as assessed by Par3 (Pard3)/CD133 (Prominin)/Actin, Zo-1/phosphorylated myosin light chain (P-MLC)/Actin, N-Cadherin (N-Cad)/CD133/Actin, and PKCξ stainings. Remnants of rosettes, cells organized in rings, can be found instead. (D,E) Another ES cell line (NERT^ΔOP^), with tamoxifen-inducible Notch1 signalling, confirms that repression of Notch signalling with DAPT reduces rosette numbers, and also reveals that activation of Notch signalling in the presence or absence of DAPT up-regulates the number of rosettes, whether Notch activity is induced on day 3 (D) or day 7 (E). Images in (A) were acquired on a fluorescence microscope at 10× magnification. Scale bars in (C) are 50 μm. Bar graphs depict means from three experiments performed in triplicate, error bars indicate standard deviation: ***significant difference at p<0.001; **significant difference at p<0.01; *significant difference at p<0.05. For separate channels please see Fig. S6.

To assess if Notch receptor activity is required for neural rosette formation, we used a pharmacological inhibitor of Notch receptor cleavage, the γ-secretase inhibitor DAPT. Treatment of *CSL^+/−^* ES cells with DAPT from day 0 or day 2 phenocopied the loss of neural rosette structures seen in *CSL^−/−^* ES cell differentiations (Fig. 4A,B). Later treatment with DAPT allowed us to assess whether Notch signaling was required also for the maintenance of existing rosettes. Treatment with DAPT on day 7 led to the breakdown of all rosette structures within 15 hours, compared to the occasional breakdown in control-treated cells (Movie S1 and S2). Treatment of *CSL^+/−^* cells with DAPT at day7 also led to a striking loss of rosette markers at day 8 (Fig. 4C and Fig. S6), and instead rings of cells could be found, resembling craters, rather than rosettes.

Activation of the Notch1 receptor was temporally controlled using the NERT^ΔOP^ cell line, in which Notch ICD is linked to a portion of the human estrogen receptor, holding Notch ICD in the cytoplasm in the absence of 4-hydroxytamoxifen (4-OHT), and relocating Notch ICD to the nucleus in the presence of 4-OHT [53]. A four hour treatment of NERT^ΔOP^ with 4-OHT on day 3 was sufficient to induce an increase in the number of rosettes seen at day 8 (Fig. 4D), and a 16 hour treatment on day 7 was also able to enhance the number of rosettes at day 8, indicating that while there may be an early effect on neural specification, a direct effect of Notch on rosette formation or stability is possible (Fig. 4E). DAPT treatment was unable to block NERT-induced rosette formation (Fig. 4D,E), since the Notch moiety in NERT is Notch ICD, which is insensitive to γ-secretase inhibitors. Collectively these results show that canonical Notch signaling is directly required for the maintenance of ES cell-derived neural rosettes and that the level of Notch signaling dynamically controls the numbers of neural rosettes.

### Notch Signaling is not Required for the Initial Stages of Neuroepithelial Induction or Acquisition of Polarity

To better understand the stage at which Notch signaling becomes critical for the neural rosette morphology we further delineated the steps of acquisition of polarity that lead to rosette formation in vitro. We monitored F-actin distribution during 6 days of wild-type ES cell differentiation and found that while F-actin was almost absent on day 1, F-actin foci appeared on day 3 (Fig. S7A, day 3, yellow arrow), followed by the appearance of epithelial-type structures with lumens (Fig. S7A, day 3, white arrow). By day 4, epithelial-type structures were abundant (Fig. S7A, day 4, white arrows) and by day 5, clear rosette structures with large lumens and dense cell nuclei were observed, (Fig. S7A, day 5 white arrow, please also see Materials and Methods for the formula used to assess lumen and colony size in conjunction with the number of cells). By day 6, large-lumen structures persisted, but small-lumen rosettes with a lower density of nuclei were also abundant (Fig. S7A, day 6 white arrow). This progression from single, unpolarized cells to mature neural rosettes, through a number of defined intermediate stages, is schematized in Fig. S7B.

**Figure 5:**
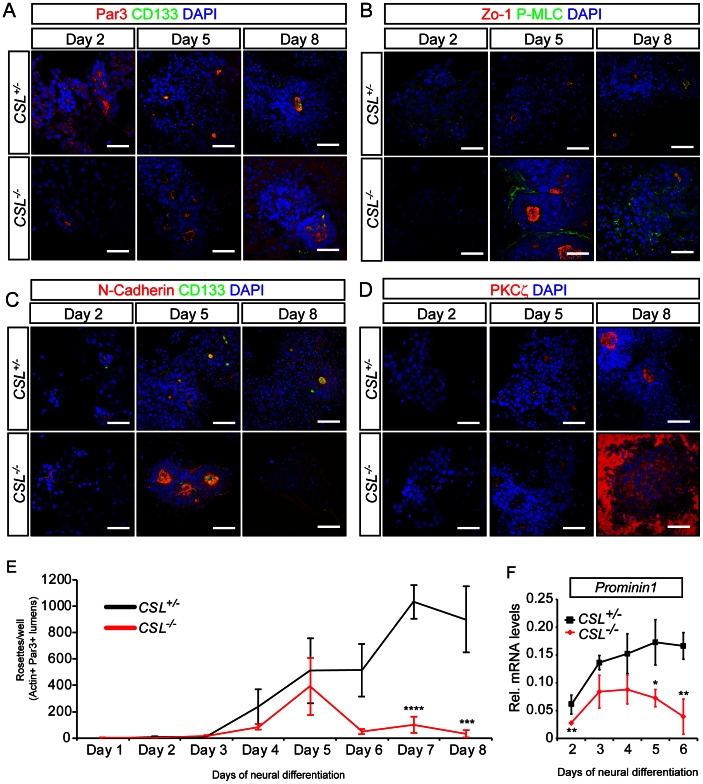
Notch signalling is not required for the acquisition of polarity and initial development of rosettes during neural differentiation. Immunohistochemistry for (A) Par3/CD133, (B) Zo-1/phosphorylated myosin light chain (PMLC), (C) N-cadherin/CD133 and (D) PKCζ reveal that up until day 5, lumen/polarity stains are similar in *CSL^+/−^* and *CSL^−/−^* differentiations, and that *CSL^−/−^* differentiations display lumens at around day 5, similar to *CSL^+/−^* differentiations. The development of these lumens is quantified in (E), showing that indeed development is similar up to day 5 (N = 3). (F) *CD133* qPCR follows a similar pattern over 6 days of differentiation, with an initially normal induction and subsequent loss of *CD133* mRNA. Error bars show standard deviation. ***significant difference at p<0.001; **significant difference at p<0.01; *significant difference at p<0.05. All scale bars are 50 μm.

We next tested the ability of *CSL^+/−^* and *CSL^−/−^* ES cells to transition through the steps of polarity formation. By day 5, both cell lines were able to form lumens, with initial accumulation of apical polarity markers and lumen formations (Fig. 5A–D). However, while *CSL^+/−^* ES cells proceeded to form large-lumen and small-lumen neural rosettes, polarity was lost in *CSL^−/−^* cells by day 8 (quantified in Fig. 5E). By 8 days of differentiation, very few organized epithelial structures were observed in the *CSL^−/−^* cultures, consistent with the severe reduction in rosette structures relative to the *CSL^+/−^* control (Fig. 3C, *CSL^+/−^*, day 5–6 white arrows). Most lumens found in *CSL^−/−^* differentiations were very large and were surrounded by a compact layer or two of cells. However, lumens in *CSL^+/−^* differentiations were smaller and the cells surrounding them were more dispersed (Fig. 5A–D and Fig. S8). Concurrent with the normal initialization of polarity and subsequent loss of polarity in *CSL^−/−^* differentiations, *Prominin1* mRNA was induced in both *CSL^+/−^* and *CSL^−/−^* differentiations, but rapidly lost in *CSL^−/−^* cells from day 4 onwards (Fig. 5F). *Prominin1* expression mimicked the previously reported pattern of *Nestin* expression [55], expressed in neural progenitors, with initially normal induction but premature decline in *CSL^−/−^* differentiations around day 4. *Pard3* expression was generally lower in *CSL^−/−^* cultures than in *CSL^+/−^* cultures while *aPKCζ*, *ZO-1*, *Rho-kinase 1/2, RhoA/E/V* and *Shroom3* showed no difference (Fig. S9 and data not shown).

In sum, these results suggest that Notch is not required for neuroepithelial induction or initial polarization, but that Notch is instead required for the maturation of polarized structures into fully developed rosettes, a process that coincides with increased *Prominin1* expression, in wild-type cells.

### Loss of Notch Signaling Accelerates Neuronal Differentiation at the Expense of Neural Stem Cell Maintenance

**Figure 6:**
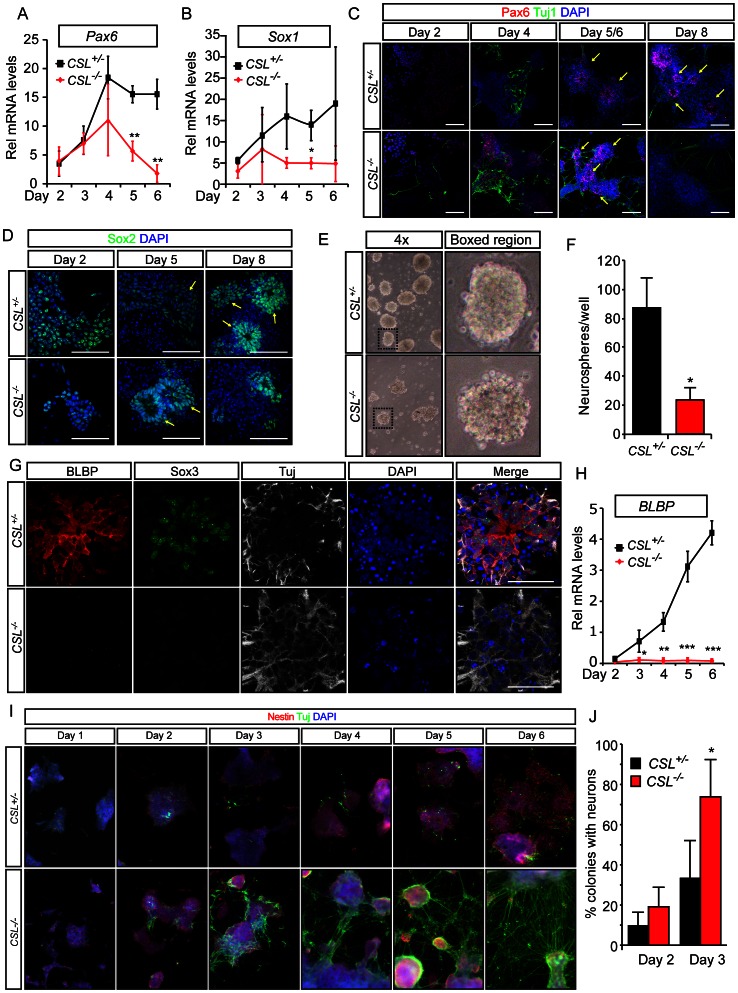
Loss of Notch signaling accelerates neuronal differentiation. Time-course analysis of Pax6 (A) and Sox1 (B) mRNA in *CSL^+/−^* and *CSL^−/−^* ES cell neural differentiations reveal normal neural induction but a rapid depletion of neuroepithelial progenitors in *CSL^−/−^* differentiations. Immunohistochemistry for Pax6, Tuj1 (C) and Sox2 (D) confirm that these markers are induced lost beginning at day 5 in *CSL^−/−^* differentiations. Note that oval structures with neural stem cells surrounding a central lumen (yellow arrows) can be found in both *CSL^+/−^* and *CSL^−/−^* cultures at day 5. (E,F) A larger number of neurospheres are derived from *CSL^+/−^* cultures after 7 days of monolayer differentiation than from *CSL^−/−^* differentiations, quantified in (F). In addition, *CSL^−/−^* spheres display an aberrant appearance with cells bulging out (boxed region in E). At day 8 (G) the neural stem cell marker Sox3 and the radial glia marker and Notch target gene BLBP are absent in *CSL^−/−^* differentiations. BLBP mRNA (H) is not detected in *CSL^−/−^* cultures over 6 days of differentiation. Finally, *CSL^−/−^* differentiations (I) lose Nestin expression and differentiate into Tuj1+ neurons more rapidly than CSL+/− differentiations, this is statistically significant (J) from day 3 of differentiation, as seen in this quantification of the number of colonies in *CSL^+/−^* and *CSL^−/−^* differentiations containing TuJ1 positive cells at day 2 and day 3. Scalebar in (C) (confocal, 5 μm section, 20×) and (D) (confocal, 2 μm section, 40×) is 100 μm, scalebar in (G) (confocal, 2 μm section, 20×) is 50 μm. Panels in (I) were taken on a fluorescence microscope at 10×. Graphs depict means from three experiments performed in triplicate, error bars indicate standard deviation: ***significant difference at p<0.001; **significant difference at p<0.01; *significant difference at p<0.05.

Notch signaling is required for induction of radial glial cell identity [56] and loss of Notch signaling induces neuronal differentiation [57]. To assess the differentiation status of the *CSL^−/−^* cultures, we examined the expression of the neuroepithelial, radial glial, and neuronal markers. The neuroepithelial genes *Sox1* and *Pax6,* and the neural stem cells marker Sox2, were induced and then lost in *CSL^−/−^* cells (Fig. 6A–C and Fig. S10) following a similar pattern to *Prominin* and *Nestin* expression (Fig. 5F and [58]). Furthermore, while neural stem cell lines could be derived from *CSL^+/−^* monolayers following the Conti. et. al. protocol [58], which selects for growth of radial glial cell-type neural stem cells, we were unable, in multiple tests, to grow neural stem cell lines from *CSL^−/−^* cultures though low numbers of neurospheres were derived in the first derivation step (data not shown). Using a simpler protocol that combines both neural progenitor and radial glial type stem cell potentials, derivation of neurospheres from day 7 differentiations revealed that the number of neurospheres from *CSL^−/−^* cultures was indeed reduced compared to *CSL^+/−^* cells (Fig. 6E, F). *CSL^+/−^* neurospheres displayed a tight, rounded, appearance, while *CSL^−/−^* spheres appeared less dense and individual cells seem to bulge out from the spheres (Fig. 6F).

At day 8, *CSL^+/−^* rosette structures displayed radiating BLBP+Sox3+ radial glia cells [58], surrounded by Tuj1+ neurons, but BLBP was not detected in *CSL^−/−^* cultures (Fig. 6G) and *BLBP* mRNA was not expressed in *CSL^−/−^* differentiations, while it was robustly expressed from day 3 in *CSL^+/−^* cultures (Fig. 6H). It should be noted however that *BLBP* is a Notch target gene [59]. Unfortunately no other radial glia-specific antibodies tested stained *CSL^+/−^* rosettes, and we are therefore unable to definitively state that radial glia are absent. Nestin expression was comparable in in *CSL^+/−^* and *CSL^−/−^* cultures until day 4 when expression began to diminish in *CSL^−/−^* cultures though continued to increase in *CSL^+/−^* cultures, concurrent with formation of Nestin+ bipolar cells (Fig. 6I and Fig. S11). These data indicate that while *CSL^−/−^* ES cells are able to form neuroepithelial tissue it is unlikely that they are able to form radial glial cells.

While few TuJ1 positive neurons were observed in day 2 *CSL^+/−^* differentiations, they were considerably more prevalent in day 2 *CSL^−/−^* cultures, a difference that increased strikingly by day 3 (Fig. 6I, J). The morphology of fully differentiated *CSL^+/−^* and *CSL^−/−^* cells was also noticeably different. By 8 days of differentiation, *CSL^−/−^* cultures displayed numerous Tuj1+ neurons with processes stretching between unpolarized neural clusters (Fig. 6I). In contrast, *CSL^+/−^* cells formed polarized colonies with radially organized Nestin+ and BLBP+ progenitors (Fig. 6G, I). Acceleration of neuronal differentiation at the expense of neural progenitors in *CSL^−/−^* differentiations was evident prior to maturation of neural epithelia into rosette structures, suggesting independent roles for Notch in regulation of neuronal differentiation and rosette maintenance.

Neurulation in vivo, and neural rosette formation in vitro, are both dependent on adequate levels of folic acid [60], [61], and folic acid is recommended almost world-wide as a daily supplement to pregnant women. Additionally, it has been suggested that folic acid acts on Notch signaling in neural cells [62]–[64] to regulate proliferation and neurogenesis. Three concentrations of folic acid failed to rescue rosettes in *CSL^−/−^* cells (Fig. S12). Both cell lines showed a dependency on folic acid, since methotrexate, a folic acid antagonist, blocked rosette or non-polarized cell cluster formation (Fig. S12), an effect which could be partially rescued with folic acid, demonstrating independent roles for Notch and folic acid in neural polarity maintenance.

### Rosette Maintenance is not a Pre-requisite for Normal Neural Differentiation

As perturbation of Notch signaling affected neural polarity maintenance, radial glial cell formation and neuronal differentiation, it is possible that Notch maintenance of polarity in neural rosettes blocks neural progenitor differentiation rather than Notch signaling acting directly on the differentiation program. This is supported by studies showing inhibition of differentiation due to ectopic expression of CD133 and Pard3 [49], [65]. To test if breaking down progenitor polarity forces neuronal differentiation, we asked whether disruption of neural rosettes in a manner distinct from Notch inhibition would affect differentiation.

**Figure 7:**
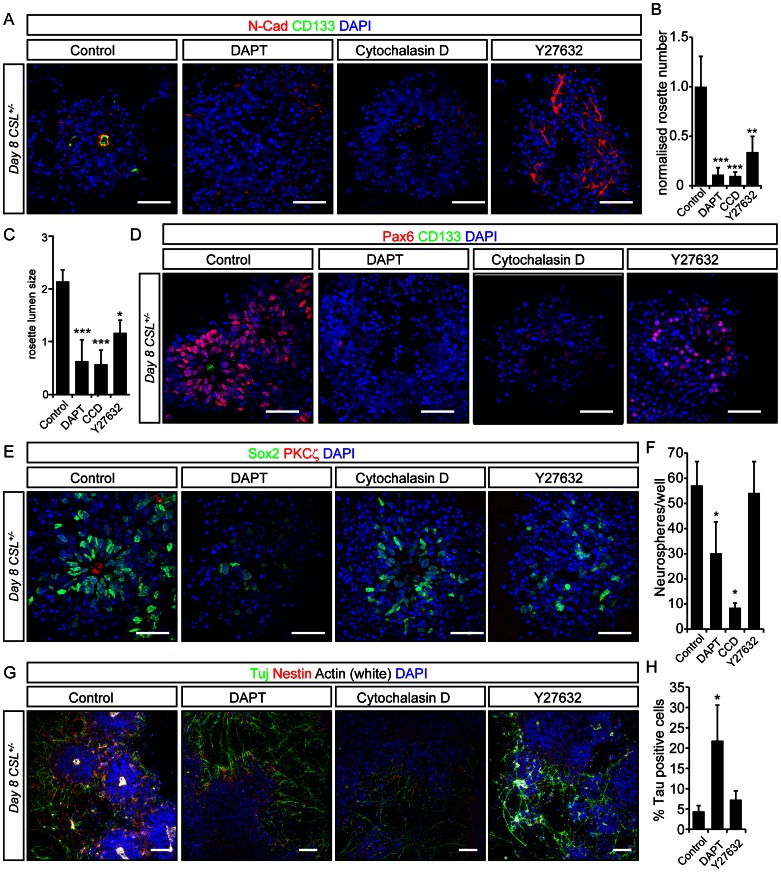
Differentiation of neural rosettes into neurons is not dependent on appropriate polarity cues in vitro. (A) Immunohistochemistry for N-Cadherin and CD133, of *CSL^+/−^* rosettes treated with the γ-secretase inhibitor DAPT, Cytochalasin D or Y27632 overnight between day 7 and day 8 depletes rosettes, leaving rings of cells lacking apical markers. Loss of rosette structures 15 hours after initiation of treatment is quantified in (B). Measure of lumen size 12 hours after initiation of treatment demonstrates a reduction in lumen size, quantified in (C). Neural stem cell markers Pax6 (D) and Sox2 (E) are dramatically reduced by DAPT treatment, but are less severely affected by Cytochalasin D or Y27632. (F) Neurospheres derived from *CSL^+/−^* cultures treated overnight between day 7 and 8 reveal that DAPT treatment and Cytochalasin D compromises neural stem cell potential, while Y27632 has no effect on stem cell potential. (G) Immunohistochemistry for Tuj1, Nestin and phalloidin staining for actin shows that DAPT redistributes actin and induces neuronal differentiation, while Y27632 and Cytochalasin D redistribute actin staining but have no discernible effect on TuJ1+ neurons. (H) The effect of loss of polarity on neuronal differentiation was quantified using a Tau-GFP ES cell line using FACS. Only DAPT induced an increase in the number of neurons 48 hours after initiation of inhibitor treatment. Please see Fig. S15 for gating. Bar graphs depict means from three experiments performed in triplicate, error bars indicate standard deviation: ***significant difference at p<0.001; **significant difference at p<0.01; *significant difference at p<0.05.

We utilized an inhibitor of actin polymerization (Cytochalasin D), or an inhibitor of Rho kinase (ROCK) activity (Y27632) to address this question. Treatment of *CSL^+/−^* ES cells with Cytochalasin D or Y27632 on day 7 of differentiation caused a redistribution of rosette markers and cell nuclei making rosettes barely distinguishable on day 8, mimicking DAPT treatment (Fig. 7A, B and Fig. S13). Cytochalasin D and Y27632 further resembled DAPT treatment, in that both led to a decrease in lumen size in the remaining rosettes (Fig. 7C). DAPT treatment greatly reduced the number of cells expressing Pax6 and Sox2, while Cytochalasin D and Y27632 mildly affected these two markers (Fig. 7 D, E and Fig. S13, 14). Using the neurosphere assay, DAPT and Cytochalasin D treatment both reduced neural stem cell potential, while Y27632 had no effect. The Cytochalasin D effect is expected as it induces cell cycle arrest. While DAPT treatment somewhat increased the number of Tuj1+ neurons in cultures after overnight treatment (Fig. 7F and Fig. S14B) and significantly increased the number of Tau-GFP (a marker of neurons) cells after 48 hours, Y27632 had no effect on the number of Tau+ neurons (Fig. 7 and Fig. S15). 48 hour treatment of the cells with Cytochalasin D was toxic to the cells, while shorter pulses showed no effect on Tau-GFP+ cell numbers (data not shown). These results suggest that while DAPT is extremely efficient at breaking down polarity and inducing neuronal differentiation, rosette maintenance itself does not directly sustain progenitors, as breaking down rosettes through actin or ROCK inhibition was not sufficient to induce neuronal differentiation in the presence of endogenous Notch signaling.

## Discussion

In this report we address the relationship between Notch signaling and apical/basal polarity in mouse early nervous system development and in ES cells undergoing neural differentiation. We find that *CSL^−/−^* mouse embryos exhibit a neurulation phenotype with aspects of polarity defects, and that neural rosette maintenance in ES cells undergoing neural differentiation is dynamically controlled by Notch signaling. Loss of Notch signaling also exerts an effect on neural differentiation in vitro, with accelerated differentiation into neurons at the expense of radial glial type neural stem cells.

Our observations from *CSL^−/−^* mouse embryos reveal that they die around E10.5, which is in accordance with the original report describing *CSL* knock-out mice [19]. We also show that the early nervous system phenotype can be subdivided into three categories, with different severities of neurulation defects, and that in the mild and intermediate categories, the neural tube was undulating. An undulating neural tube has been observed when genes required for apical/basal functions have been ablated [29]. This suggested that Notch may be linked to cell polarity control, and we indeed observed a reduction in expression of the apical marker CD133/prominin in both *CSL^−/−^* embryos and in *CSL^−/−^* ES cell neural differentiations. Our data, from both the in vivo situation and from neural differentiation in ES cells, demonstrate a critical role for both CSL- and gamma secretase-dependent canonical Notch signaling in neural polarity maintenance. In this regard, it is interesting to note that studies from other model systems have implicated non-canonical, non-CSL-dependent, Notch signaling in control of cellular polarity and dorsal closure. For example, in *Drosophila*, dorsal closure is dependent on membrane-bound Notch, which represses signals through JNK, and is independent of CSL [66], while in zebrafish, Notch maintains apical/basal neural polarity, in a CSL-independent manner, together with the polarity proteins Crumbs and Moe [36]. It is possible that the canonical effect we see is specific to mammals, though conditional knockout of *Notch1* or *CSL* would allow a more reasonable comparison to *Drosophila* and zebrafish data.

During neural differentiation of ES cells, Notch signaling dynamically regulated neural rosette maintenance. Loss or inactivation of Notch signaling led to ablation of neural rosettes, whereas elevated Notch signaling resulted in an increase in the number of neural rosettes. This suggests that the level of Notch signaling is critical for apical/basal organization, and is in keeping with the exquisite dosage sensitivity that is characteristic for Notch signaling (see [67], [68] for review). The observation that γ-secretase inhibitors impair neural rosette maintenance is in keeping with previous reports from monolayer and embryoid body cultures [12], [16], [69], and our results extend these observations and show, for the first time, that it is CSL-mediated, receptor-dependent canonical Notch signaling that mediates this effect.

Our data show that the Notch effect on neural rosette maintenance occurs after Notch affects neural differentiation. We find that *CSL^−/−^* ES cells initiated neural differentiation and polarity programs in a similar manner to *CSL^+/−^* cells, but that from day 3, *CSL^−/−^* cells showed accelerated neuronal differentiation, at the expense of radial glial type neural stem cells and loss of polarized organization. The accelerated neuronal differentiation in the absence of Notch signaling is in contrast to a previous report, which also assessed the potential of *CSL*-deficient ES cells for neural differentiation [50]. In that report, loss of Notch, either in the absence of *CSL* or with γ-secretase inhibitor treatment, was reported to inhibit neural differentiation in favor of non-neural fates. We show here that there is a density-dependence for neural and neuronal differentiation in *CSL^−/−^* cultures, with a propensity for non-neural differentiation at high seeding densities, and pronounced accelerated neuronal differentiation at lower seeding densities, most likely due to BMP-mediated density dependent neural induction blockade [51], consistent with the role of Notch in *Drosophila* [70]. Importantly, the data from *CSL^−/−^* ES cells under lower seeding densities are in accordance with the in vivo situation, in which Notch maintains the neural stem cell state and inhibits neuronal differentiation [57].

The fact that perturbation of Notch signaling affected both neural differentiation and neural rosette maintenance left open the possibility that maintenance of polarity was important for the timing of neural differentiation. To learn whether this was the case we disrupted neural rosettes in an alternate manner, independent of Notch inhibition. Blocking actin polymerization or ROCK activity rapidly eroded neural rosettes, which is in keeping with the importance of actin cytoskeleton integrity and dynamics in the developing neural tube [7], [9], [10]. Inhibition of actin and ROCK activity, however, did not affect neuronal differentiation suggesting that polarity and differentiation are independent. It is also worth noting that disrupting rosettes with DAPT, or inducing rosette formation by inducing NICD translocation required less than 24 hours of treatment to exert an effect, while a significant difference in the number of neurons could only be seen after two days of treatment with DAPT, also indicating that the effect of Notch on polarity occurs prior to, and perhaps separately from, its effect on neurogenesis.

One explanation for the effect of Notch loss of function in rosettes is that Notch signaling, required for radial glial cell development [56], [57], [71], contributes to formation and maintenance of radial glial cells which may provide structural integrity to rosettes. Pard3 and CD133 apical component targeting is important for regulating the timing of differentiation in vivo [37], [47], [72] and basal Notch signaling components in radial glial cells are important for their self-renewal. We show that Notch is important for normal regulation of Pard3 and CD133 and is also important for establishment and maintenance of radial glial cells. This may demonstrate why loss of apical domains through Rock and actin inhibition is not sufficient to drive differentiation. We show that ablated Notch signaling diminishes *Pard3*, known to regulate apical constriction through ROCK [73]. An intriguing possibility is the idea of a positive feedback loop maintaining Notch signaling and polarity components, since Pard3 also positively regulates Notch by inhibiting Numb and Numblike, two negative regulators of Notch [37]. Another interesting possibility is that Notch, in mammals, would associate with Crumbs2, as it can with zebrafish Crumbs [36] though this mechanism may involve non-canonical Notch signaling. Crumbs has been reported to localize at the apical side of neural rosettes [18] and at the apical sides of the neuroepithelium [74]–[76]. It is required for apical cellular constriction in tracheal cells [77], and mimics Notch signaling in that it increases the number of rosettes, maintains neural stem cells in a proliferative state and inhibits neuronal differentiation [18]. In fact, loss of Crumbs2 even reduces the expression of Pard3 [18], offering yet another layer of crosstalk of Notch and polarity components to investigate further.

We observed two distinct types of rosettes in culture that can be distinguished with a simple formula based on nuclei density, lumen size and rosette size. One type, which appeared at around day 3, was characterized by cell-dense rosettes with large, hollow lumens. The second type contained fewer cells, which radiated from a small central lumen. Data from Elkabetz et. al. suggest that lumen size decreases as symmetrically dividing cells begin to undergo asymmetric differentiating divisions [16]. Together these data suggest that our large lumen rosettes represent a neuroepithelial progenitor (NEP) stage of neural tube closure and that the small lumen type represents the later radial glial phase established upon closure and initiation of neurogenesis. The effects of Notch signaling in each phase of this in vitro morphogenesis will be interesting to explore in vitro with the assistance of pharmacological agents and live imaging.

In conclusion, our data shed new light on the role of Notch signaling in cell polarity and neural rosette maintenance. As neural rosettes share morphology, topology and marker gene expression with the early neural tube [13], this information may provide insights into roles for Notch signaling in cell polarity and apical/basal organization, which may help us better understand the mechanisms underpinning folic acid independent neural tube closure defects in humans, including spina bifida and anencephaly.

## Supporting Information

Figure S1 **Notch signalling and apical markers CD133, F-actin and Zo-1 in the developing neural tube.** (A) Sections of neural tube of E8.75 *CSL^+/+^* and *CSL^-/-^* embryos, in which neurulation has completed, stained for CD133, F-actin and Zo-1 reveal a decrease in apical staining. Scale bar is 50 µm.
(TIF)

Figure S2 **Proteins localized to the apical side of the neural tube label the apical lumens of developing rosettes.** (A) Rosettes derived in ES cell culture are 3-dimensional structures with a Par3-positive central lumen. (B) Other stains which label the apical neural tube at E9.5 and the lumen of neural rosettes during ES cell neural differentiations include CD133 (Prominin), ZO-1, PKCζ, and actin. (C) Similar to the neural tube, the central lumen is surrounded by Sox3+ Pax6+ progenitors, while Tuj1+ neurons are found further away, at the periphery of the rosette.
(TIF)

Figure S3 **Notch is expressed in ES cells undergoing neural differentiation.** Separate channels for Figure 2A.
(TIF)

Figure S4 **Notch signalling is required for the presence of neural rosettes at Day 8 of differentiation.** Separate channels for Figure 4C shown here. (A) *CSL^+/-^* and *CSL^-/-^* ES cells were differentiated for 8 days under neural differentiation conditions. *CSL^-/-^* differentiations do not contain rosettes, as assessed by staining for rosette lumen-specific markers (A) Par3 (Pard3)/CD133 (Prominin)/Actin, (B) ZO-1 / phosphorylated myosin light chain (P-MLC) /Actin, (C) N-Cadherin / CD133Actin, and (D) PKCζ stainings. Scale bar is 50 µm.
(TIF)

Figure S5 **Differences between** ***CSL^+/-^*** **and C** ***SL^-/-^*** **cultures.** Phalloidin staining for actin and DAPI nuclear staining show striking differences in the appearance of cultures of *CSL^+/-^* and *CSL^-/-^* cells after 8 days of differentiation. While neural rosettes with radially organized cells are easily seen in *CSL^+/-^*differentiations (white arrow), these are not seen in *CSL^-/-^*differentiations, which instead contain clusters of unpolarized cells and sheets of large flat cells. Images acquired at 10×.
(TIF)

Figure S6 **Notch is required for rosette maintenance**. Separate channels for (A) Figure 4A, and (B–E) Figure 4C. (A) *CSL^+/-^* ES cells, grown under neural differentiation conditions, treated with the µ-secretase inhibitor DAPT from Day 2 or Day 6 display far fewer rosettes by day 8, as assessed by staining for DAPI and actin (A). Images acquired at 10×. (B–E) Acute treatment of *CSL^+/-^* cells with DAPT for 16 hours between Day 7 and Day 8 leads to a break-down of existing rosettes, as assessed by (B) Par3 (Pard3)/CD133 (Prominin)/Actin, (C) ZO-1 / phosphorylated myosin light chain (P-MLC) /Actin, (D) N-Cadherin (N-Cad) / CD133Actin, and (E) PKCξ stainings. Scale bar is 50 μm.
(TIF)

Figure S7 **Wildtype characterisation of timescale of polarity and rosette formation.** (A) (A) Phalloidin staining of F-actin in the first 6 days of 46C neural monolayer differentiation. Loci of actin accumulation can be seen as early as day 3 (yellow arrow) along with few epithelial structures (white arrow). More epithelial structures and the beginnings of lumens can be seen around day 4 (white arrows). Well organised rosette structures with large lumens and high cellular density appear around day 5 (arrow). While large lumen/high cell density rosettes remain, a second distinct type of rosette with small lumens and low cell density begins to appear around day 6 (arrow). Images were acquired on a fluorescence microscope at 10x magnification. (B) The progression of actin accumulation in foci to the development of rosettes is schematized here.
(TIF)

Figure S8 **Figure 5** **. Notch signalling is not required for the acquisition of polarity and initial development of rosettes during neural differentiation.** Separate channels for Figure 5 A-D.
(TIF)

Figure S9 **Par3 levels are lower, while Shroom3 and PKCζ expression are unaffected by loss of Notch signaling.** mRNA expression of (A) *Shroom3*, (B) *PKCζ* and (C) *Pard3* during 6 days of *CSL^+/-^* versus *CSL^-/-^* ES cell neural differentiation reveals no significant differences in *Shroom3* or *PKCζ*, and an overall decrease in *Pard3*.
(TIF)

Figure S10 **Loss of Notch signaling accelerates neuronal differentiation. Separate channels for** **Figure 6C and D** **.**
(TIF)

Figure S11 **Loss of Notch signaling accelerates neuronal differentiation. Separate channels for** **Figure 6** **I.**
(TIF)

Figure S12 **Folic acid cannot rescue rosettes in** ***CSL^-/-^*** **differentiations.** Three concentrations of folic acid (FA) from day 1 of neural differentiation failed to rescue rosettes in Day 8 neural differentiations of *CSL^-/-^* ES cells. For *CSL^+/-^* cells, rosettes were counted, and for *CSL^-/-^* cells non-polarised cell clusters were counted. No neural rosettes could be found in *CSL^-/-^* differentiations. Both rosettes and cell clusters were ablated when the cells were treated with the folic acid antagonist methotrexate (MTX), an effect which could be partially rescued with folic acid.
(TIF)

Figure S13 **Separate channels for** **Fig 7** **A and D.**
(TIF)

Figure S14 **Separate channels for** **Fig 7** **E and G.**
(TIF)

Figure S15 **Gating strategy for Tau+ cells.**
(TIF)

Movie S1 DAPT Rosette structures in *CSL^+/-^* ES cells, undergoing neural differentiation and which are treated with DAPT on day 6, break down within 15 hours of treatment. Images were acquired at 37 degrees Celsius with a 10× objective every 20 min using the Image Xpress Micro.
(WMV)

Movie S2 Control Control movie of untreated cells.
(AVI)
