# Supplementary figures and images for: Notch Signaling Maintains Neural Rosette Polarity
Source: PLoS One. 2013 May 10;8(5):e62959. doi: 10.1371/journal.pone.0062959 (PMC3651093; doi:10.1371/journal.pone.0062959)

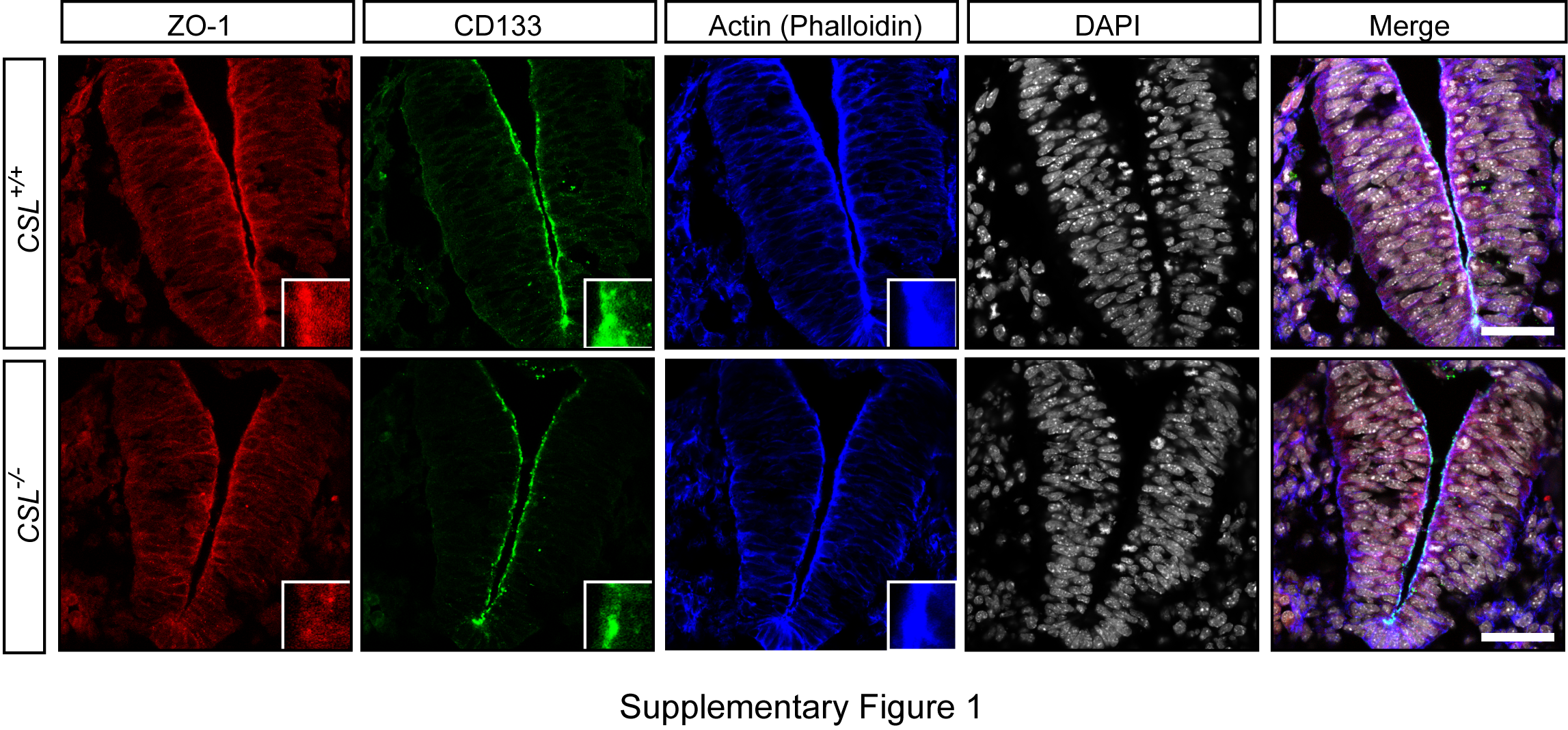

Supplement: Figure S1 — Notch signalling and apical markers CD133, F-actin and Zo-1 in the developing neural tube. (A) Sections of neural tube of E8.75 CSL+/+ and CSL-/- embryos, in which neurulation has completed, stained for CD133, F-actin and Zo-1 reveal a decrease in apical staining. Scale bar is 50 µm. (TIF) [file pone.0062959.s001.tif]

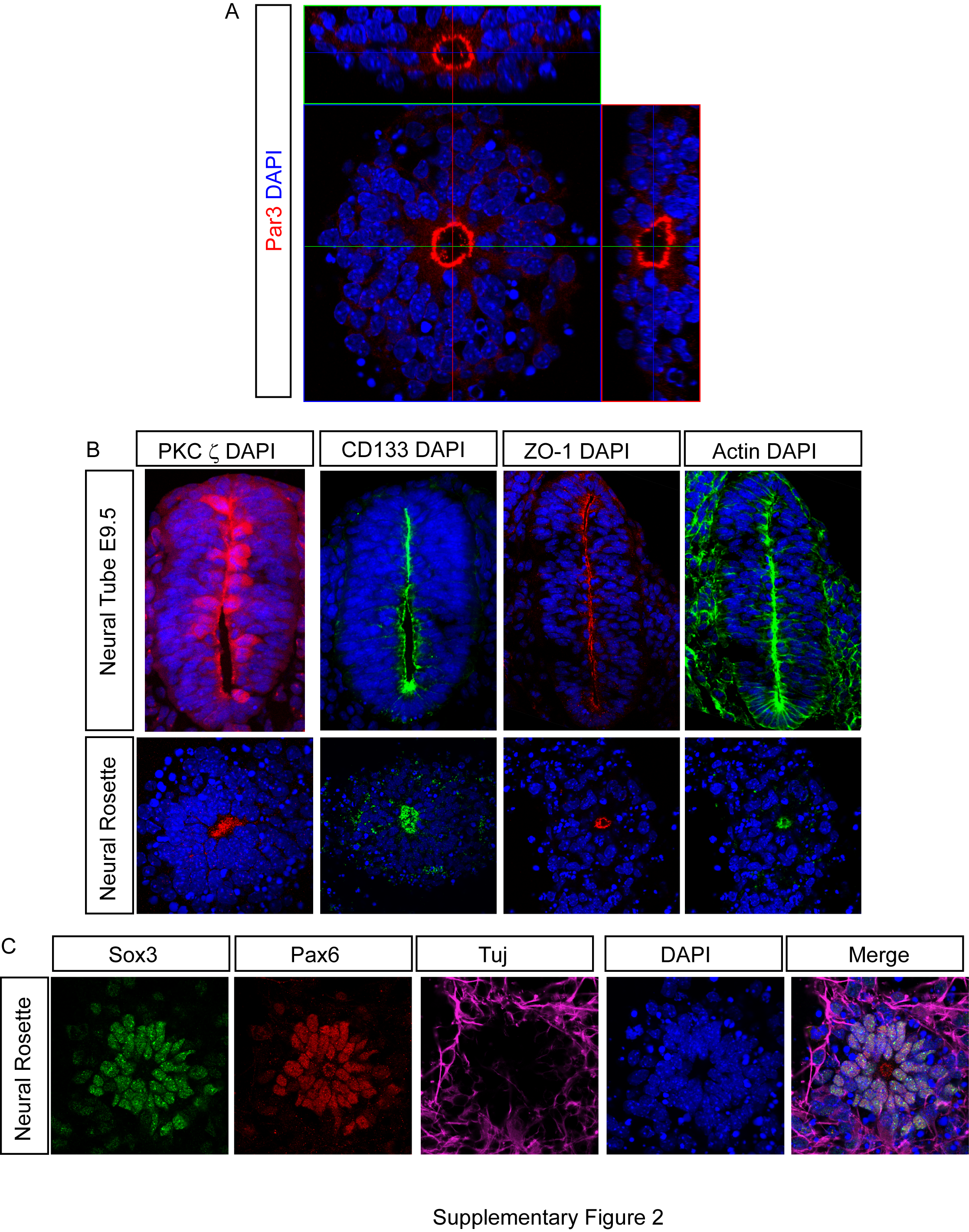

Supplement: Figure S2 — Proteins localized to the apical side of the neural tube label the apical lumens of developing rosettes. (A) Rosettes derived in ES cell culture are 3-dimensional structures with a Par3-positive central lumen. (B) Other stains which label the apical neural tube at E9.5 and the lumen of neural rosettes during ES cell neural differentiations include CD133 (Prominin), ZO-1, PKCζ, and actin. (C) Similar to the neural tube, the central lumen is surrounded by Sox3+ Pax6+ progenitors, while Tuj1+ neurons are found further away, at the periphery of the rosette. (TIF) [file pone.0062959.s002.tif]

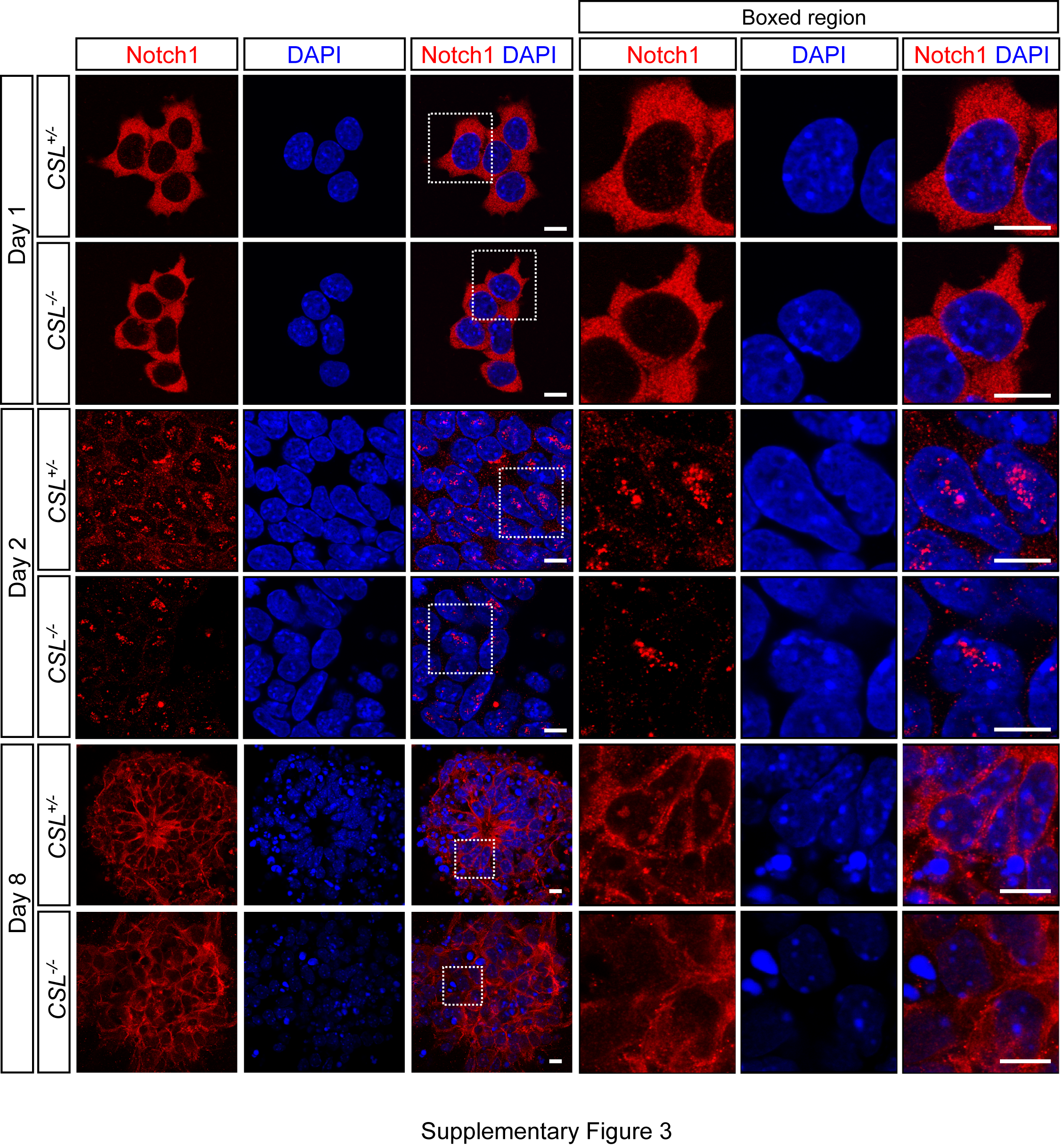

Supplement: Figure S3 — Notch is expressed in ES cells undergoing neural differentiation. Separate channels for Figure 2A. (TIF) [file pone.0062959.s003.tif]

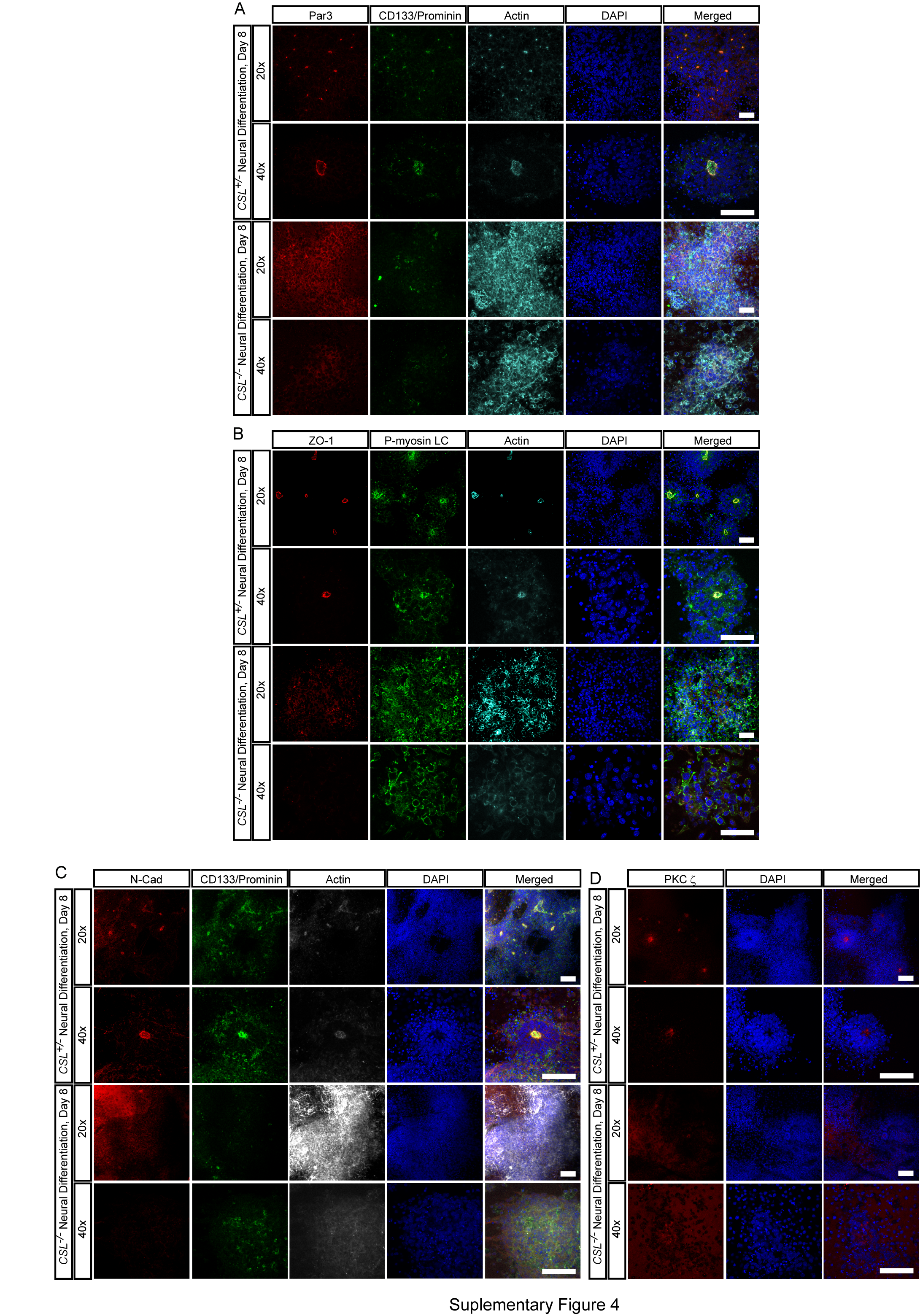

Supplement: Figure S4 — Notch signalling is required for the presence of neural rosettes at Day 8 of differentiation. Separate channels for Figure 4C shown here. (A) CSL+/- and CSL-/- ES cells were differentiated for 8 days under neural differentiation conditions. CSL-/- differentiations do not contain rosettes, as assessed by staining for rosette lumen-specific markers (A) Par3 (Pard3)/CD133 (Prominin)/Actin, (B) ZO-1 / phosphorylated myosin light chain (P-MLC) /Actin, (C) N-Cadherin / CD133Actin, and (D) PKCζ stainings. Scale bar is 50 µm. (TIF) [file pone.0062959.s004.tif]

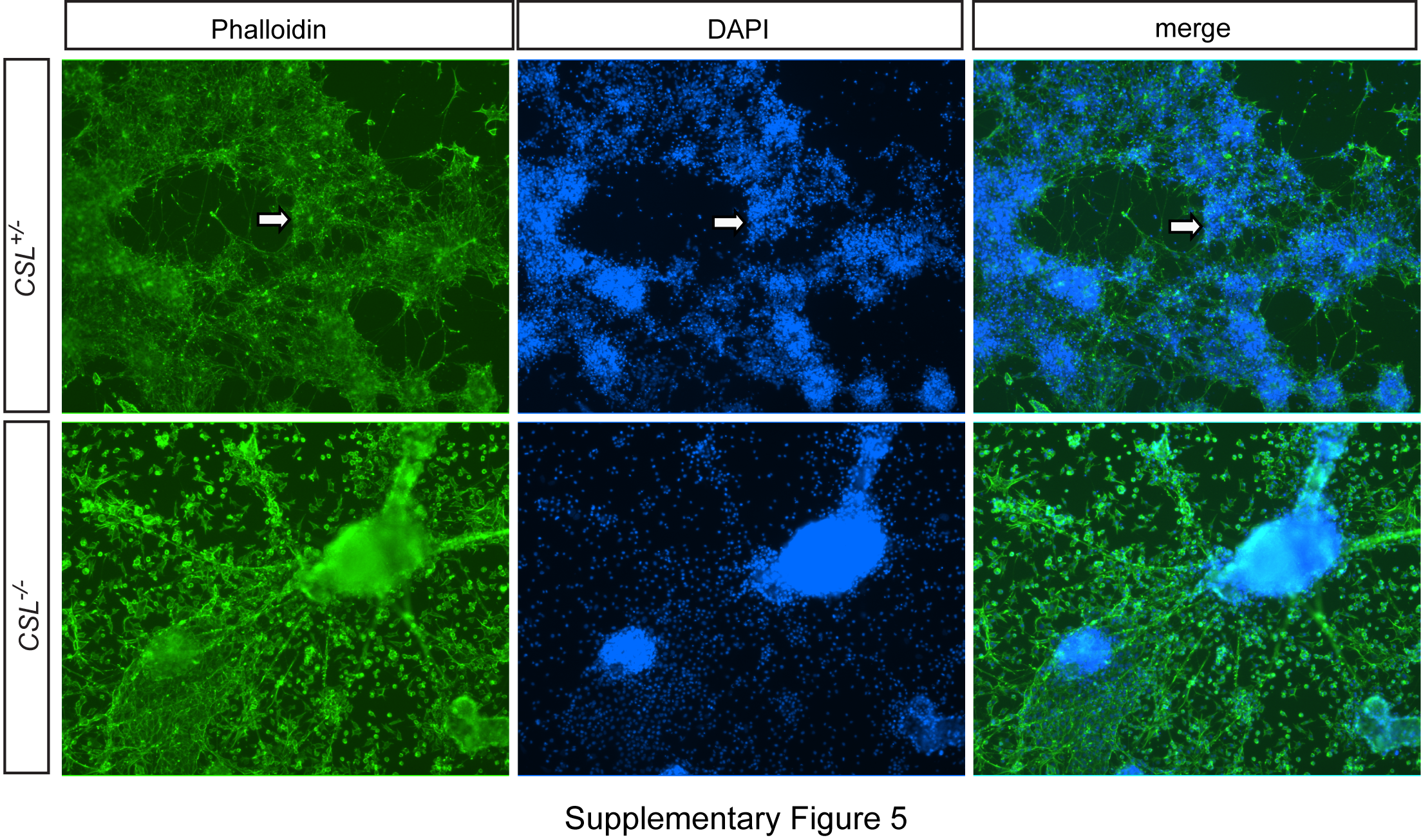

Supplement: Figure S5 — Differences between CSL+/- and C SL-/- cultures. Phalloidin staining for actin and DAPI nuclear staining show striking differences in the appearance of cultures of CSL+/- and CSL-/- cells after 8 days of differentiation. While neural rosettes with radially organized cells are easily seen in CSL+/-differentiations (white arrow), these are not seen in CSL-/-differentiations, which instead contain clusters of unpolarized cells and sheets of large flat cells. Images acquired at 10×. (TIF) [file pone.0062959.s005.tif]

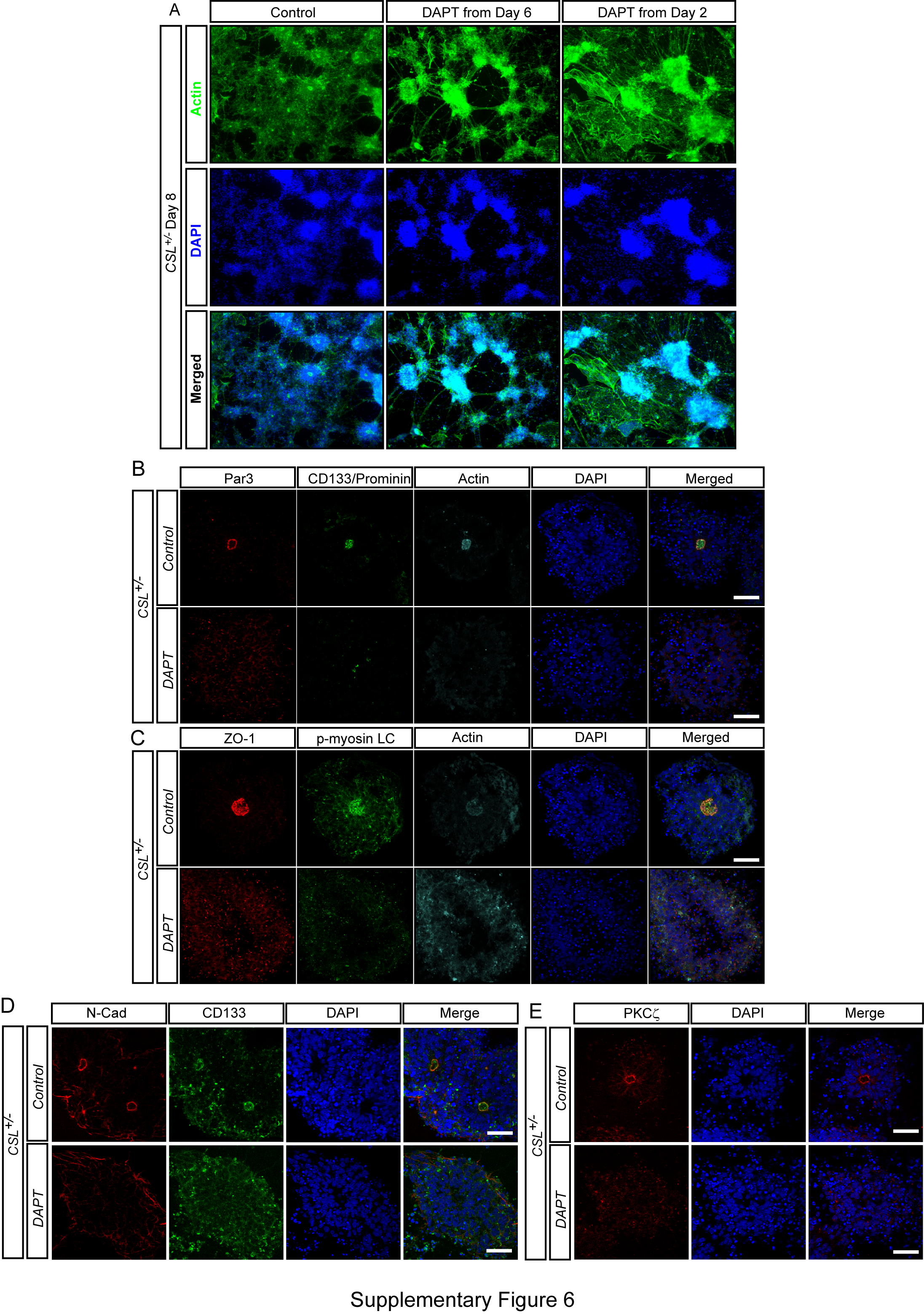

Supplement: Figure S6 — Notch is required for rosette maintenance. Separate channels for (A) Figure 4A, and (B–E) Figure 4C. (A) CSL+/- ES cells, grown under neural differentiation conditions, treated with the µ-secretase inhibitor DAPT from Day 2 or Day 6 display far fewer rosettes by day 8, as assessed by staining for DAPI and actin (A). Images acquired at 10×. (B–E) Acute treatment of CSL+/- cells with DAPT for 16 hours between Day 7 and Day 8 leads to a break-down of existing rosettes, as assessed by (B) Par3 (Pard3)/CD133 (Prominin)/Actin, (C) ZO-1 / phosphorylated myosin light chain (P-MLC) /Actin, (D) N-Cadherin (N-Cad) / CD133Actin, and (E) PKCξ stainings. Scale bar is 50 μm. (TIF) [file pone.0062959.s006.tif]

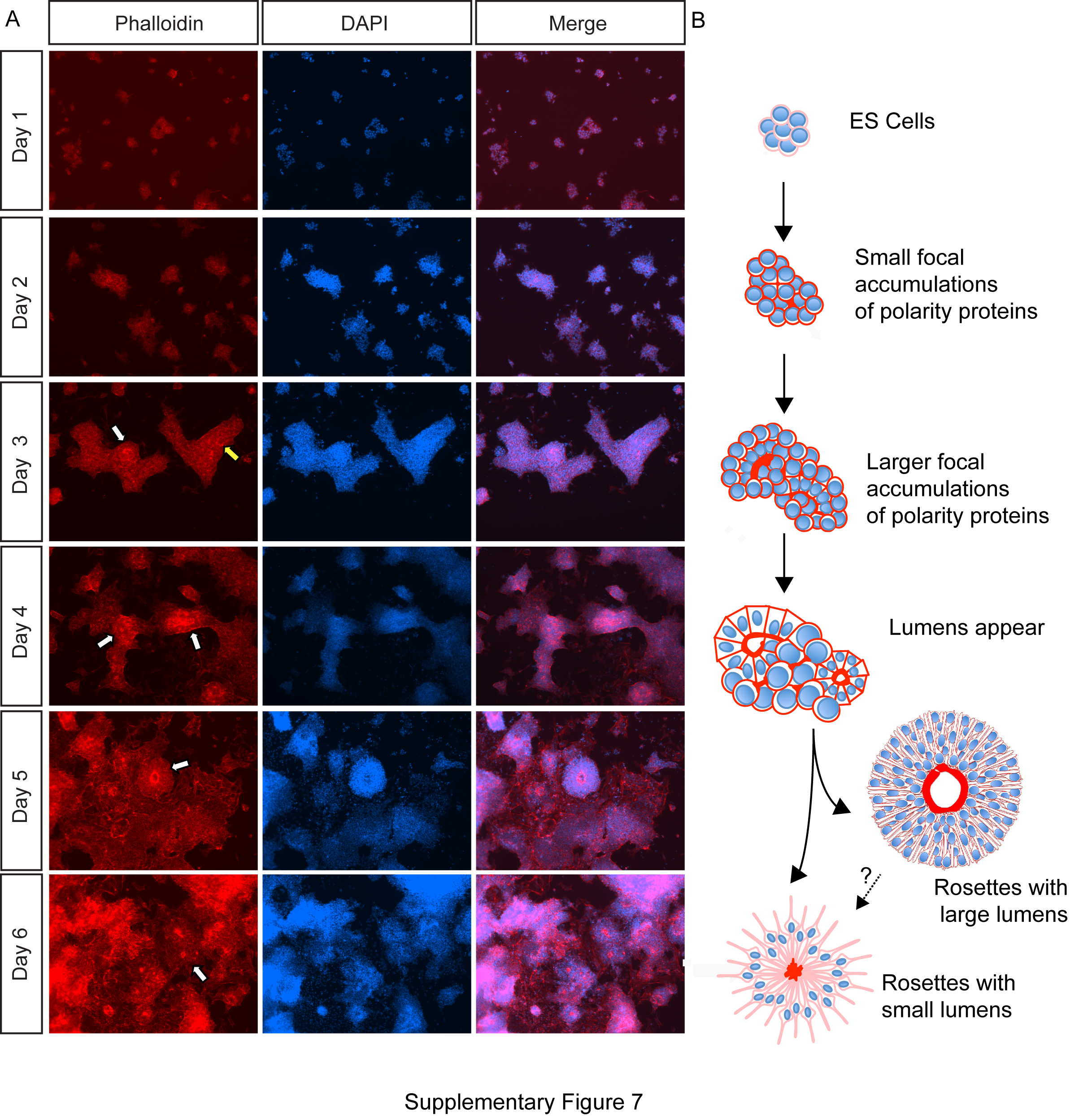

Supplement: Figure S7 — Wildtype characterisation of timescale of polarity and rosette formation. (A) (A) Phalloidin staining of F-actin in the first 6 days of 46C neural monolayer differentiation. Loci of actin accumulation can be seen as early as day 3 (yellow arrow) along with few epithelial structures (white arrow). More epithelial structures and the beginnings of lumens can be seen around day 4 (white arrows). Well organised rosette structures with large lumens and high cellular density appear around day 5 (arrow). While large lumen/high cell density rosettes remain, a second distinct type of rosette with small lumens and low cell density begins to appear around day 6 (arrow). Images were acquired on a fluorescence microscope at 10x magnification. (B) The progression of actin accumulation in foci to the development of rosettes is schematized here. (TIF) [file pone.0062959.s007.tif]

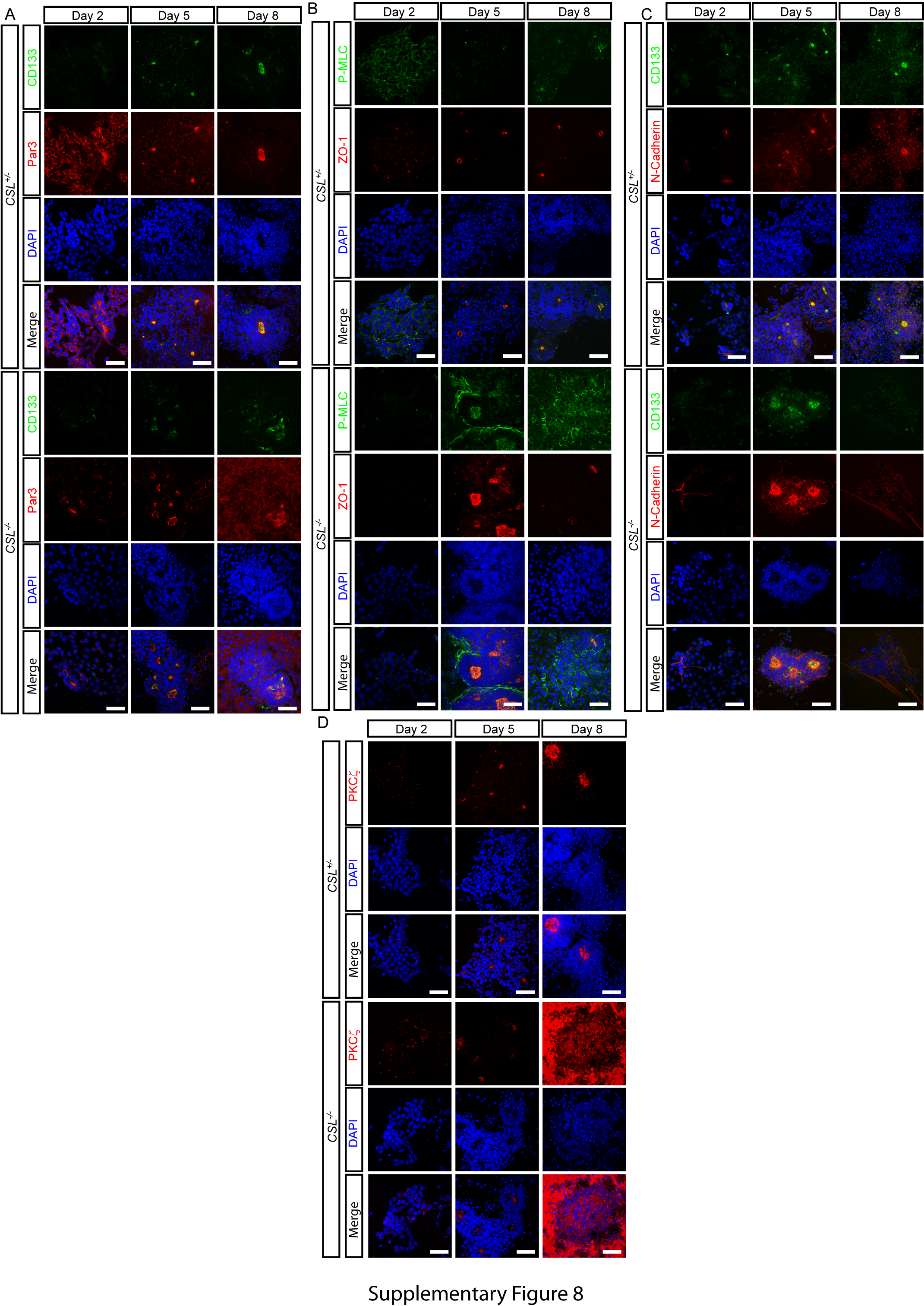

Supplement: Figure S8 — Figure 5 . Notch signalling is not required for the acquisition of polarity and initial development of rosettes during neural differentiation. Separate channels for Figure 5 A-D. (TIF) [file pone.0062959.s008.tif]

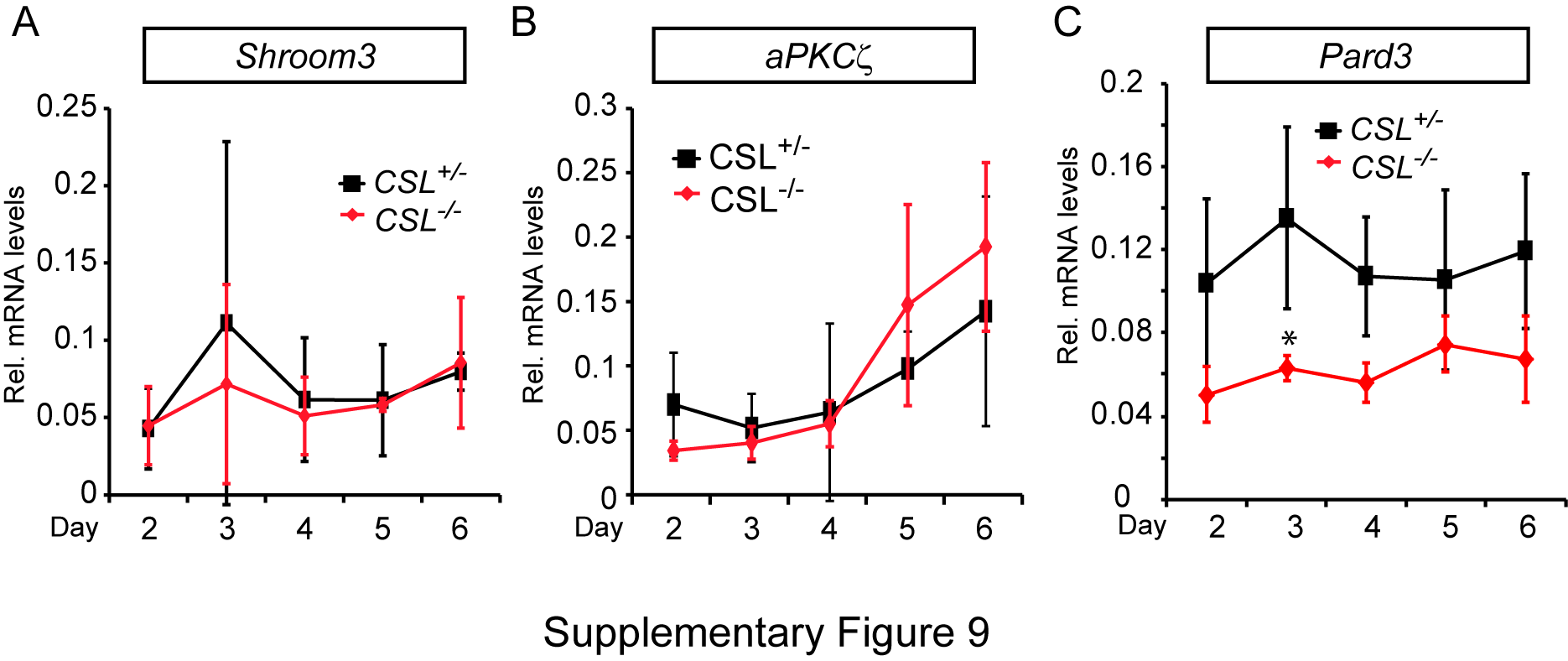

Supplement: Figure S9 — Par3 levels are lower, while Shroom3 and PKCζ expression are unaffected by loss of Notch signaling. mRNA expression of (A) Shroom3, (B) PKCζ and (C) Pard3 during 6 days of CSL+/- versus CSL-/- ES cell neural differentiation reveals no significant differences in Shroom3 or PKCζ, and an overall decrease in Pard3. (TIF) [file pone.0062959.s009.tif]

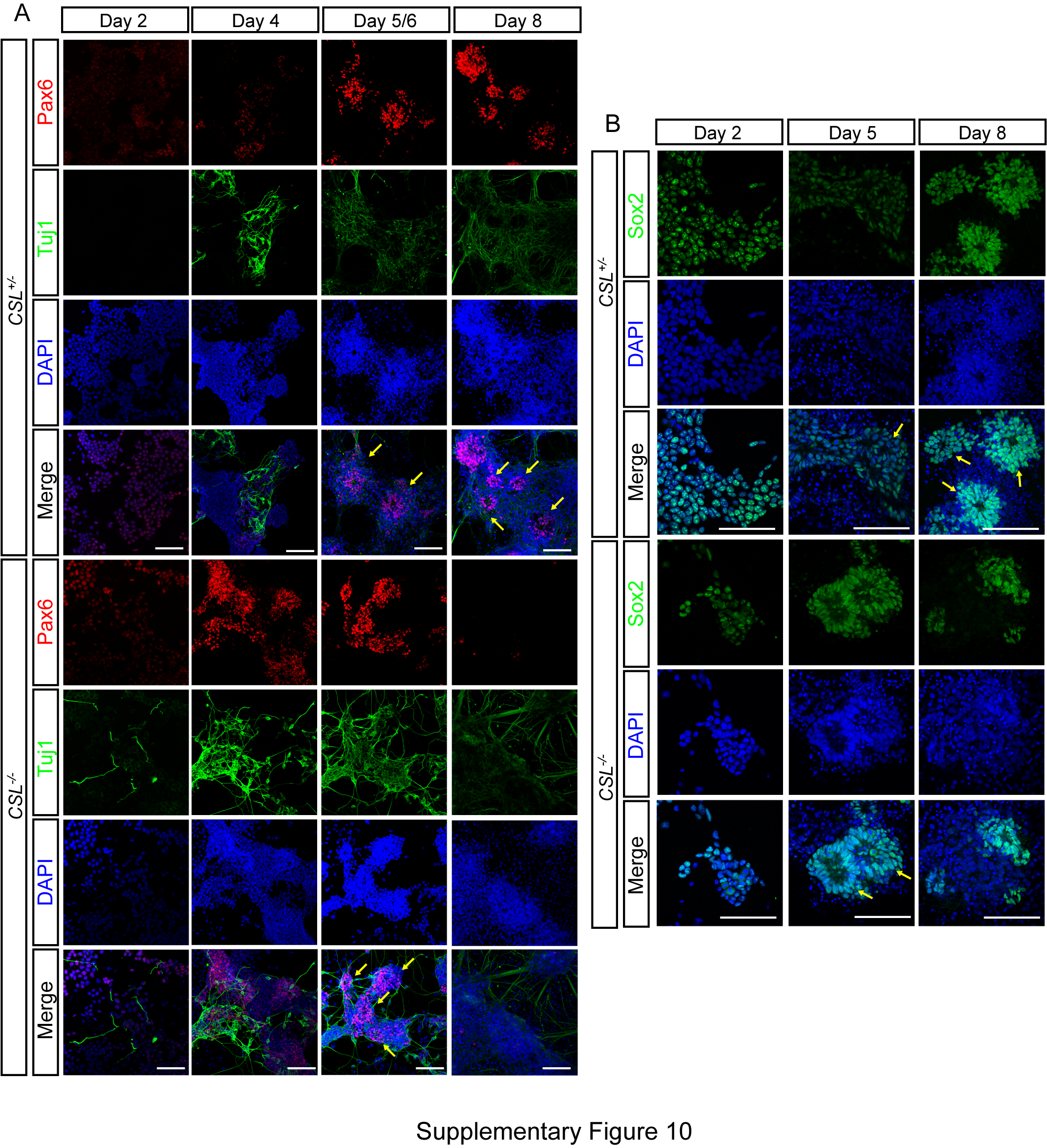

Supplement: Figure S10 — Loss of Notch signaling accelerates neuronal differentiation. Separate channels for Figure 6C and D . (TIF) [file pone.0062959.s010.tif]

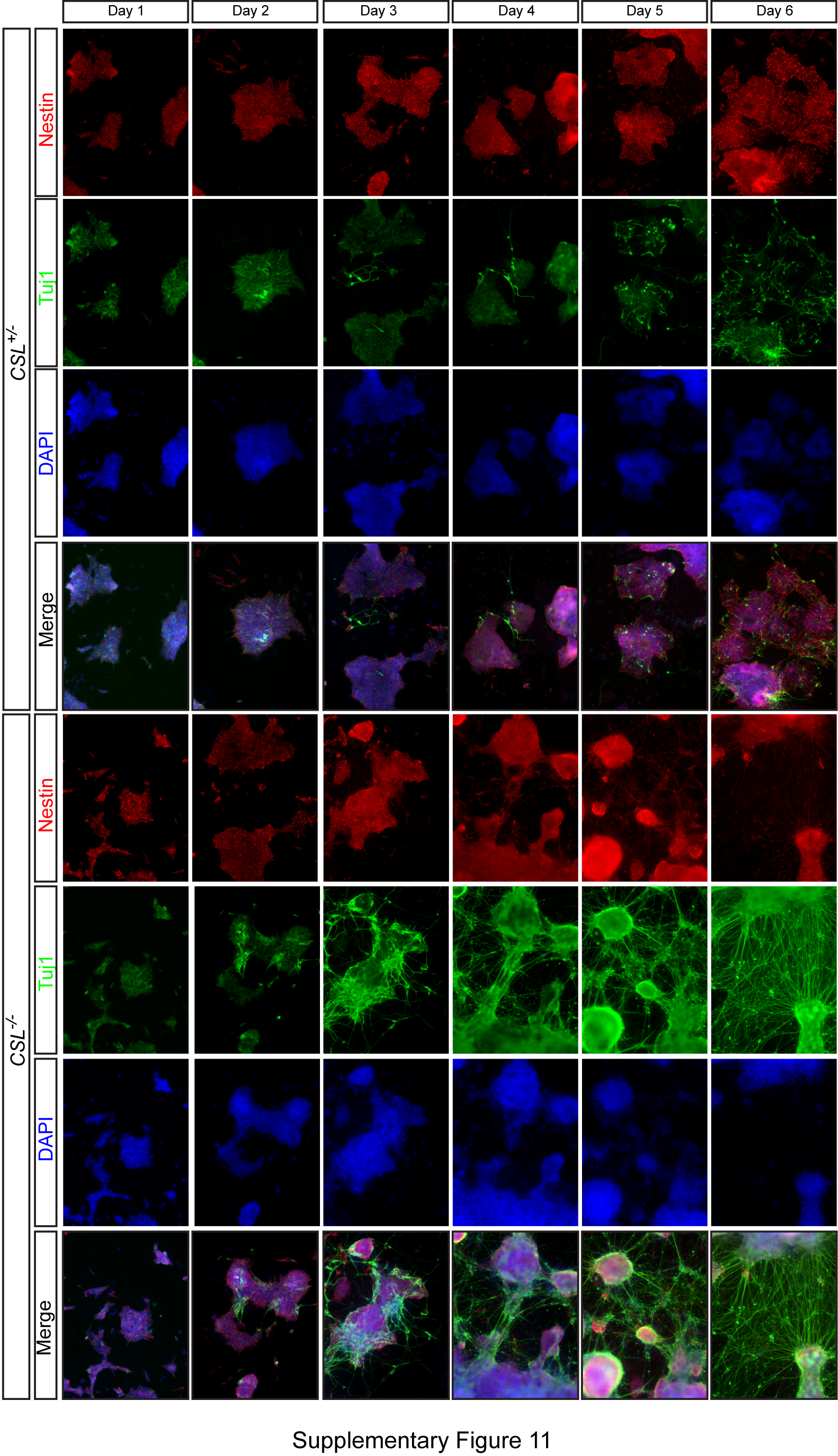

Supplement: Figure S11 — Loss of Notch signaling accelerates neuronal differentiation. Separate channels for Figure 6 I. (TIF) [file pone.0062959.s011.tif]

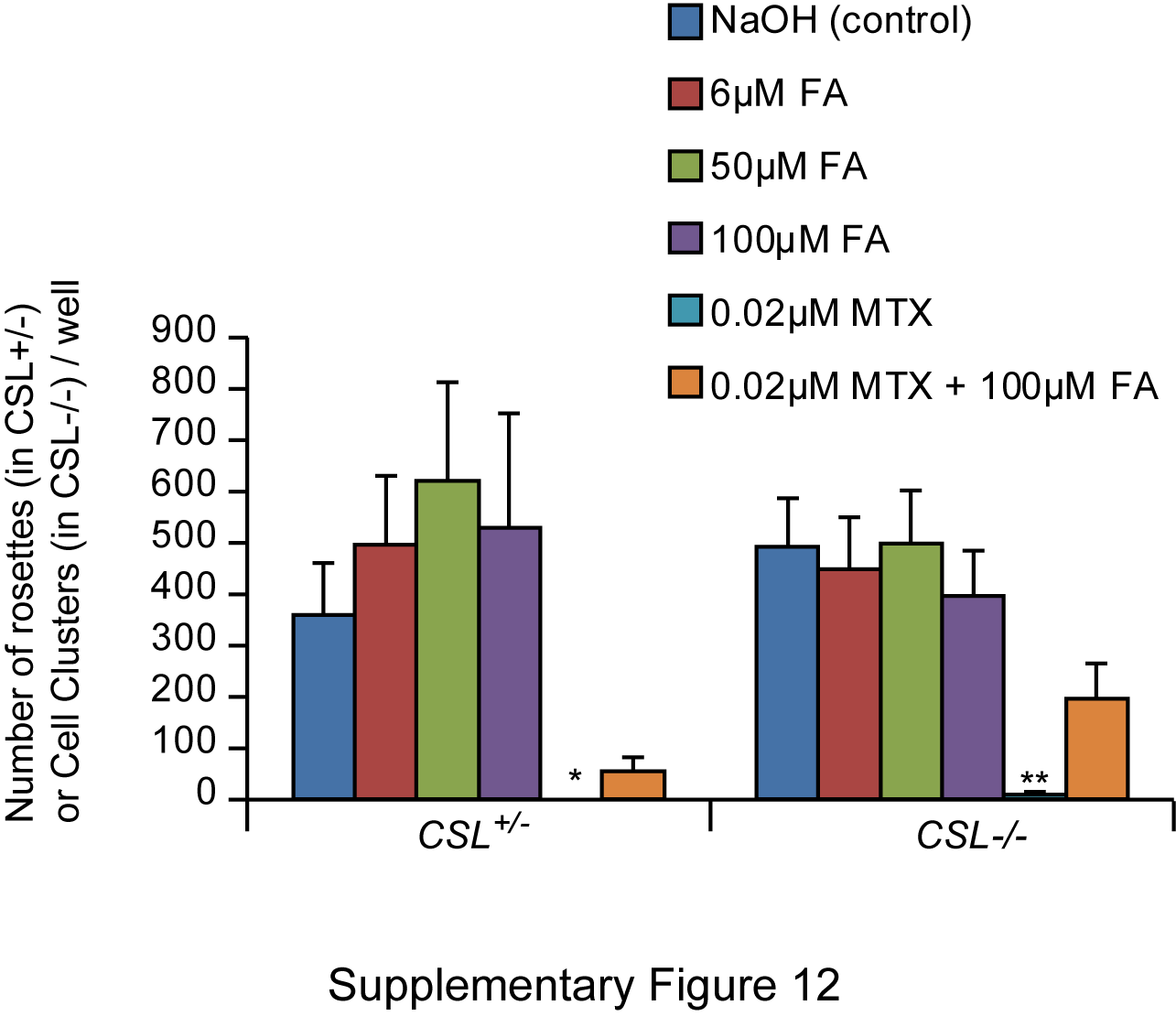

Supplement: Figure S12 — Folic acid cannot rescue rosettes in CSL-/- differentiations. Three concentrations of folic acid (FA) from day 1 of neural differentiation failed to rescue rosettes in Day 8 neural differentiations of CSL-/- ES cells. For CSL+/- cells, rosettes were counted, and for CSL-/- cells non-polarised cell clusters were counted. No neural rosettes could be found in CSL-/- differentiations. Both rosettes and cell clusters were ablated when the cells were treated with the folic acid antagonist methotrexate (MTX), an effect which could be partially rescued with folic acid. (TIF) [file pone.0062959.s012.tif]

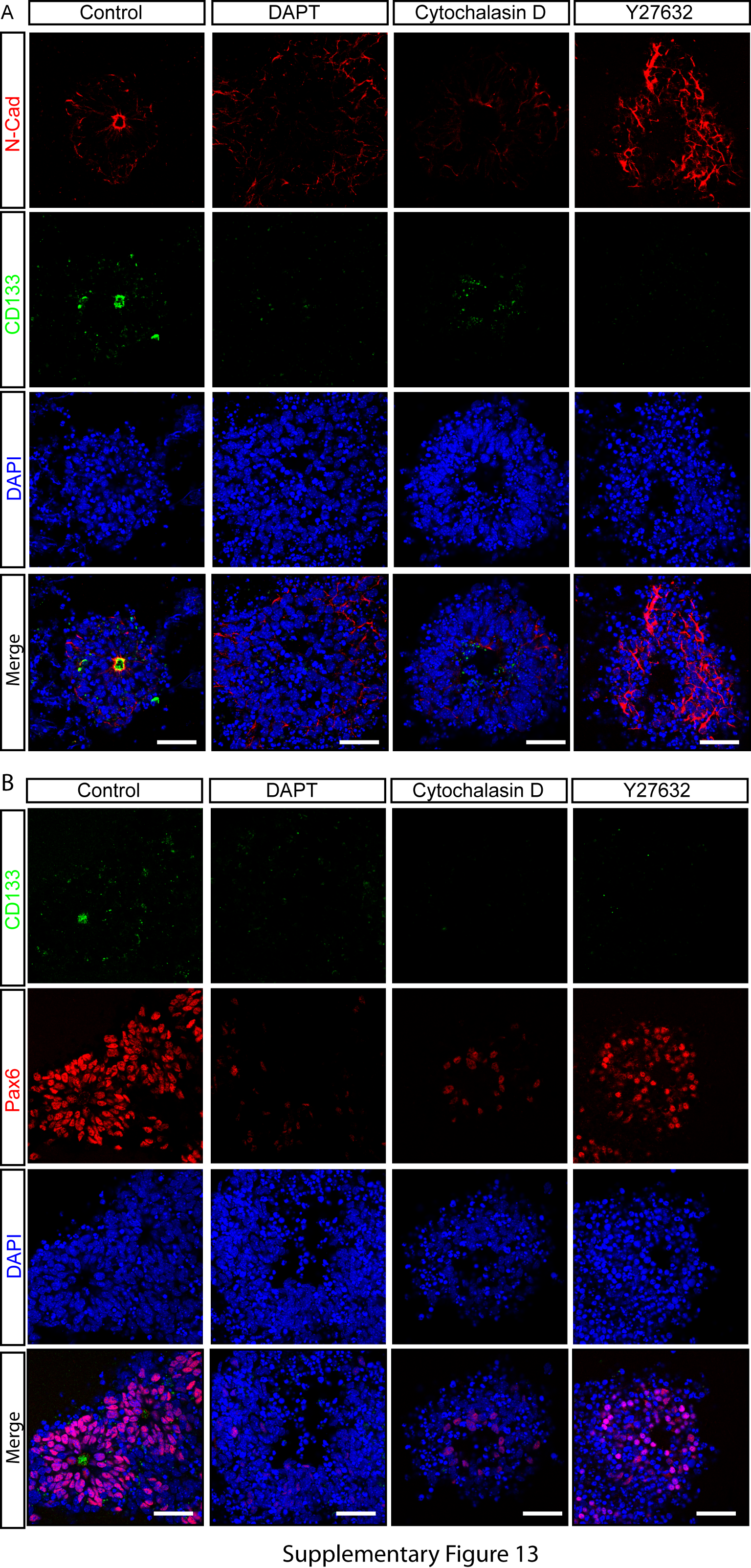

Supplement: Figure S13 — Separate channels for Fig 7 A and D. (TIF) [file pone.0062959.s013.tif]

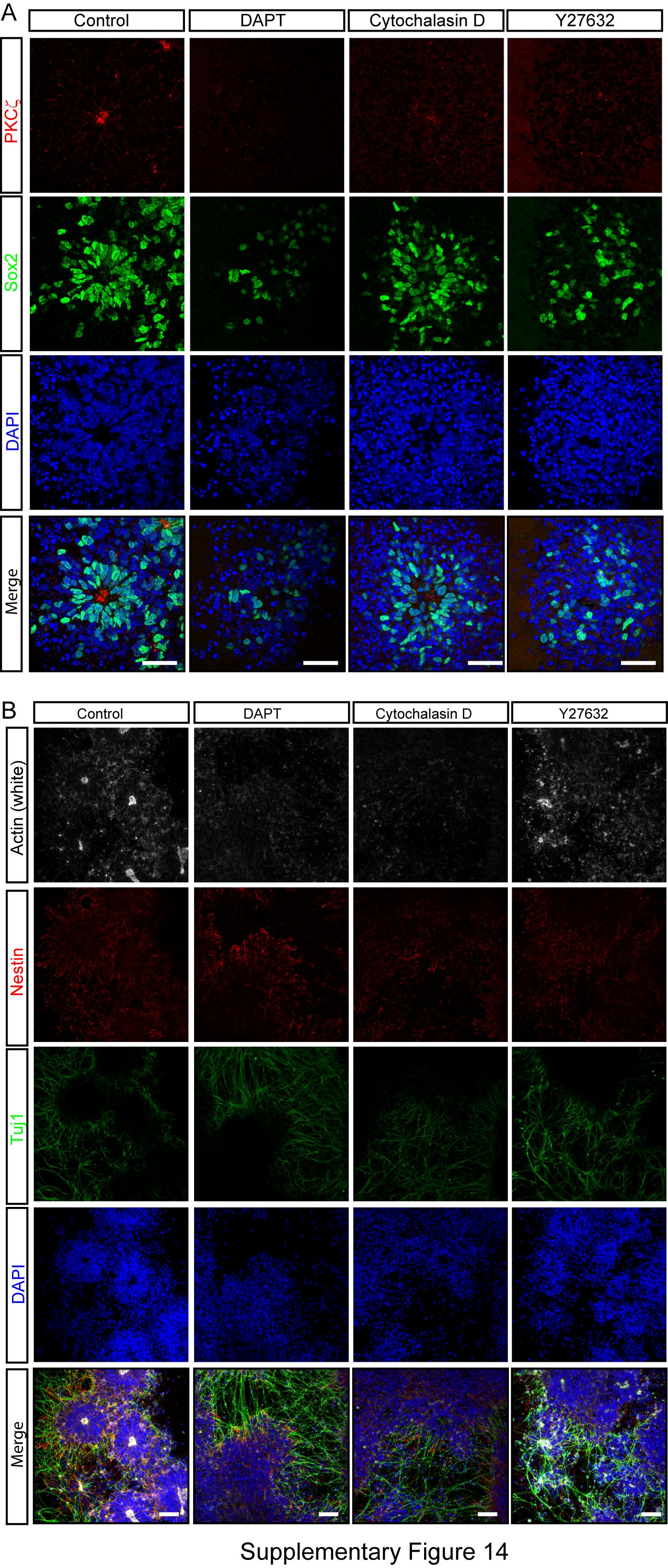

Supplement: Figure S14 — Separate channels for Fig 7 E and G. (TIF) [file pone.0062959.s014.tif]

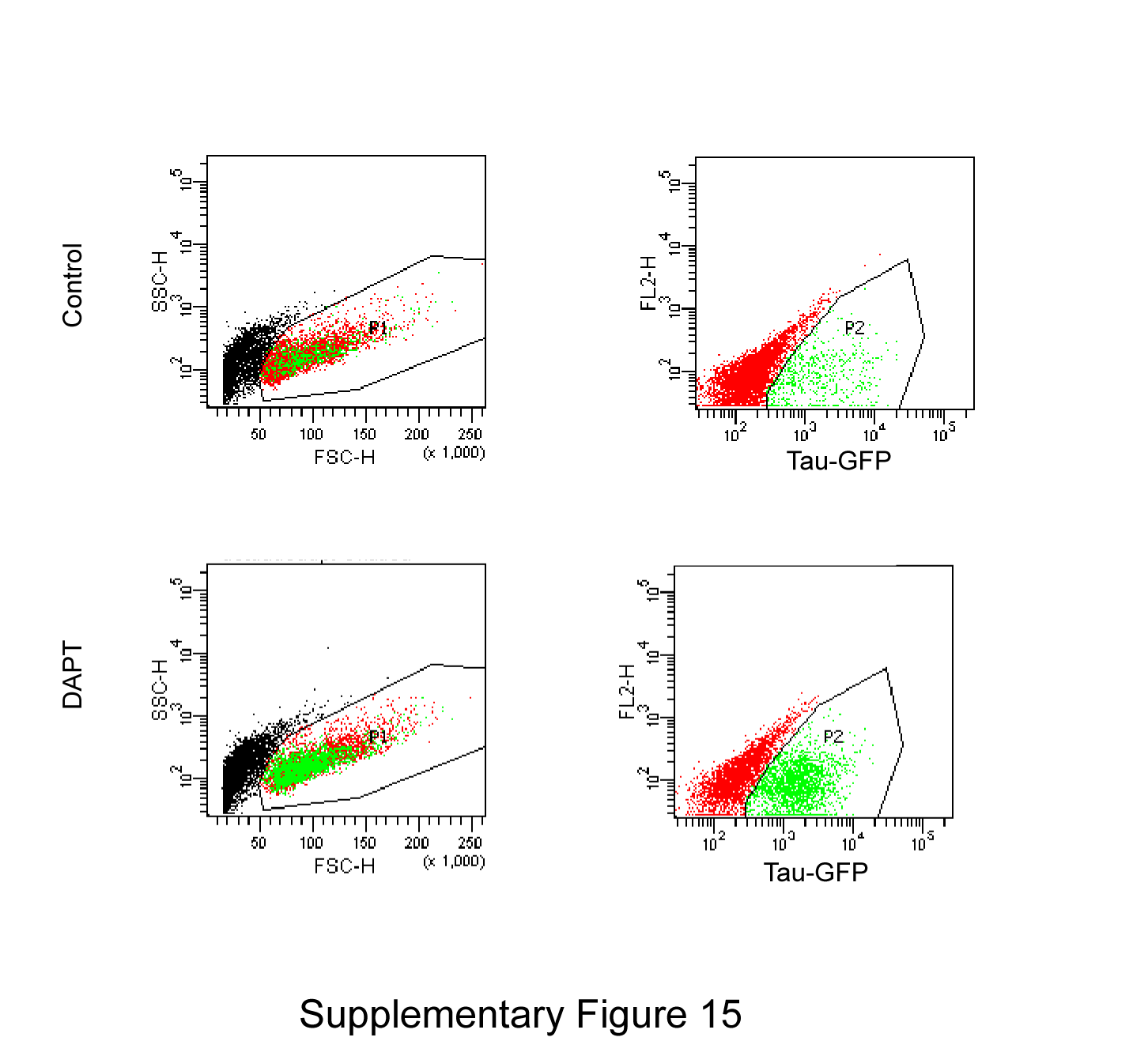

Supplement: Figure S15 — Gating strategy for Tau+ cells. (TIF) [file pone.0062959.s015.tif]
